# Investigation of the protein profile of silkworm (*Bombyx mori*) pupae reared on a well-calibrated artificial diet compared to mulberry leaf diet

**DOI:** 10.7717/peerj.6723

**Published:** 2019-06-12

**Authors:** Cristina Lamberti, Francesco Gai, Simona Cirrincione, Marzia Giribaldi, Micol Purrotti, Marcello Manfredi, Emilio Marengo, Benedetto Sicuro, Alessio Saviane, Silvia Cappellozza, Maria Gabriella Giuffrida, Laura Cavallarin

**Affiliations:** 1 Institute of Science of Food Production, National Research Council, Grugliasco, Italy; 2 Research Centre for Engineering and Agro-Food Processing, Council for Agricultural Research and Analysis of Economics, Torino, Italy; 3 Center for Translational Research on Autoimmune and Allergic Diseases (CAAD), Novara, Italy; 4 Department of Veterinary Sciences, University of Turin, Grugliasco, Italy; 5 Centre of Research for Agriculture and Environment, Sericulture Laboratory, Council for Agricultural Research and Analysis of Economics, Padova, Italy

**Keywords:** Insect, Farming, Artificial diet, Mass spectrometry

## Abstract

**Background:**

Silkworm pupae is the main by-product of the sericulture industry with an interesting nutritional profile, especially in terms of proteins. In consideration of its possible use as a food or food ingredient in Western countries, a comparative proteomic experiment has been performed to investigate the differences of the protein profile of male and female silkworm pupae reared on mulberry leaves or on an artificial diet.

**Methods:**

The nutritional profile of lyophilized silkworm pupae in terms of dry matter and ash was evaluated according to the AOAC procedures, the total nitrogen content was determined by a nitrogen analyzer and the silkworm pupae gross energy value was measured using an adiabatic calorimetric bomb. The comparative proteomic analysis was performed on male and female silkworm pupae reared on mulberry leaves or on the artificial diet. Proteins were separated by two-dimensional electrophoresis and, after a multivariate statistical analysis, the differentially expressed proteins were identified by LC-MS/MS.

**Results:**

The comparative proteomic approach highlighted 47 silkworm pupae proteins differentially expressed comparing diet and gender. PCA analysis showed that seven proteins were more effective in discriminating the sex and five were more effective in discriminating the diet type. In spite of the above-mentioned differences in the silkworm pupae protein profile, no strong alteration of the pupa physiological traits have been demonstrated, suggesting a general silkworm pupae flexibility to adapt to a well-balanced artificial diet. Differences in lipid transport and metabolism were found among the experimental groups, that might have a relevant effect on the timing and on hormone secretion. This aspect may also affect silk production, as univoltine strains are the most productive. The proteomic data provided in this work, may offer a contribution in understanding also the influence of gender and farming strategy on the allergen profile of *Bombyx mori*, when used as food or as a food ingredient. Female silkworm pupae reared on mulberry leaves seemed to contain lower levels of known allergens than those reared in the other experimental conditions; these findings will have to be taken into account when farming *B. mori* for food production purposes. However, our results need to be supported by further characterization of the allergenic potential of *B. mori*.

## Introduction

Silkworms (*Bombyx mori*) are insects that are able to convert plant proteins to produce silk, and, while silkworm pupae is considered the main by-product of the sericulture industry. Silkworm pupae has been used as a food, a medicine and as an animal feed in many Asian countries for a long time (Dong et al., 2017), due to its interesting nutritional profile, in terms of protein, fat, and chitin contents.

Traditionally, silkworm larvae are fed with fresh mulberry leaves, which are also their natural diet. However, in order to obviate the serious drawbacks of mulberry leaves, such as the seasonal limitation concerning the supply of fresh leaves, the possible harm from parasites or pesticides and the high labor costs, different artificial diets containing essential nutrients have been studied (Cappellozza et al., 2005; Dong et al., 2017; Zhou et al., 2008). These artificial diets may affect the larval mortality and/or the length of the larval cycle to various extents, and the resulting silk production is often slightly reduced (Cappellozza et al., 2005). Another important aspect of the silkworm farming is related to gender. Male and female silkworm have shown different silk production abilities, in particular as far as the quality and quantity of silk are concerned. Gender has also been found to affect the growth rate at various larval stages, possibly due to a difference in nutrient utilization by the midgut, as reported by Qin et al. (2014).

Owing to the increasing interest in insects as a new food and feed protein source over the last few years, EFSA issued a scientific opinion on the topic in October 2015. They highlighted that a specific risk assessment should be performed, taking into account the whole production chain from farming to consumption, including the species to raise and the substrate to use as well as the methods for farming and processing (EFSA Scientific Committee, 2015). Among the edible insect candidates, with the greatest potential for use as food on the EU market, the silkworm seems a promising candidate from a nutritional point of view (EFSA Scientific Committee, 2015).

In light of these considerations, we designed a comparative proteomic study to characterize the protein profile of male and female silkworm pupae reared on two diets, in order to identify any possible differences in protein expression, for obtaining basic understanding of how to optimize the rearing strategies for the use of this edible insect in the food and feed sector.

## Materials and Methods

### Experimental animals

The larvae of hybrid silkworm strain (four-way polyhybrid (57 × 76)–(76 × 57) belonging to the germplasm collection of the CREA Research Centre for Agriculture and Environment (CREA-AA)) were reared under the same environmental conditions (temperature (25 ± 1 °C) and relative humidity), but on two different diets: mulberry leaves (L) or an artificial diet (A) according to Cappellozza et al. (2005) (Table S1). Seven days after reaching the cocoon stage, the pupae were harvested and sexed according to their morphological features. A vertical line across the center of the ventral side of the eighth segment and a genital aperture in the ninth sternum were considered to identify females (F), whereas only the presence of an aperture situated at the ninth sternum was used to identify males (M).

All the analyses were carried out on three batches for each treatment: males reared on A (M_A_) and on L (M_L_), and females reared on A (F_A_) and on L (F_L_).

### Nutritional profile analysis

In order to evaluate the nutritional profile of lyophilized silkworm pupae, the dry matter (DM) (#930.15) and the ash (#924.05) were assessed according to the AOAC procedures (AOAC International, 2003). The total nitrogen (N) content was determined using a nitrogen analyzer (Rapid N III; Elementar Analysen system GmbH, Hanau, Germany) according to the Dumas method and the gross energy was measured using an adiabatic calorimetric bomb (C7000; IKA, Staufen, Germany).

### Proteomic analysis

The comparative proteomic analysis was performed on three extraction replicates for each biological replicate and for each experimental condition (for a total of 36 two-dimensional electrophoresis (2DE) gels).

### Preparation of the soluble protein extracts

A pool of 10 frozen pupae (−80 °C), corresponding to 0.5 g, pulverized by means of a mincer, was solubilized in 1.5 mL of PBS (0.1M, pH 7.4) and a Complete™ (Sigma-Aldrich S.r.l., St. Louis, MO, USA) protease inhibitor was added (one tablet per 50 mL extraction solution). Each sample was sonicated on ice, under agitation, for a total of 30 s, for seven cycles, with 30 min of break after each cycle. After centrifugation (13,201×*g*, 4 °C, 10 min), the upper phase and the pellet were discarded and the supernatant protein content was determined by means of the 2D-Quant-kit (GE Healthcare, Chicago, IL, USA).

### Two-dimensional electrophoresis

Each protein extract (50 μg) was diluted in an appropriate volume of IPG rehydration buffer (7M urea, 2M thiourea, 66 mM DTT, 4% CHAPS, 0.5% ampholytes) and loaded on immobilized pH gradient strips (seven cm, linear pI gradient from 3 to 10) (Bio-Rad Italia, Hercules, CA, USA). The IPG strips were actively rehydrated for 6 h at 50 V and 20 °C, and isoelectrofocusing was carried out on a Protean IEF Cell (Bio-Rad, Hercules, CA, USA), starting with a voltage of 200 V for 1 h, then 1,000 V for 1 h and finally up to 4,000 V for a total of 25,000 Vh. The focused strips were incubated at RT in a reduction buffer (6M urea, 30% v/v glycerol, 2% w/v SDS, 50 mM Tris–HCl, pH 8.6, 2% w/v DTT) for 15 min and then in an alkylation buffer (6M urea, 30% v/v glycerol, 2% w/v SDS, 50 mM Tris–HCl, pH 8.6, 4.5% w/v iodoacetamide) for 15 min in the dark. The equilibrated strips were then embedded at the top of LDS precast homogeneous gels (NuPAGE 10% Bis–Tris, Invitrogen Corporation, Carlsbad, CA, USA) and electrophoretic separation was performed in an XCell SureLock Mini-Cell System (Invitrogen, Carlsbad, CA, USA) at RT, 200 V constant, 125 mA, 100 W for 45 min. The gels were stained with Colloidal Coomassie Blue (Candiano et al., 2004) and scanned with a ChemiDoc MP System densitometer (Bio-Rad, Hercules, CA, USA) at the resolution of 600 dpi.

### Image analysis

The image analysis was performed with PDQuest Advanced 2D Gel Analysis Software (Bio-Rad, Hercules, CA, USA). Spot detection was automatically performed using the software algorithm and the spots were verified manually. After the insertion of an appropriate number of user seeds, the matching was performed automatically and then checked manually. To ensure normalization of the spot quantities, the protein spot densities were normalized (%V) on total volumes of all the spots in each gel image.

### Mass spectrometry protein identification

The protein spots selected as being differentially expressed, excised from fresh 2DE gels, were destained overnight with 40% ethanol/50 mM NH_4_HCO_3_, washed three times with 25 mM NH_4_CO_3_ and three times with acetonitrile (ANC) and then dried in Eppendorf Concentrator 5301 (Eppendorf, Hamburg, Germany). The proteins were in-gel digested with 75 ng/μL of sequencing-grade, modified porcine trypsin (Promega, Madison, WI, USA). The peptide digests were desalted on a Discovery^®^ DSC-18 solid phase extraction 96-well plate (25 mg/well) (Sigma-Aldrich Inc., St. Louis, MO, USA), prior to mass spectrometry analysis. The LC-MS/MS analyses were performed by means of a micro-LC Eksigent Technologies (Dublin, OH, USA) system, which included a micro LC200 Eksigent pump with a 5–50 μL flow module and a programmable autosampler CTC PAL with a Peltier unit (1.0–45.0 °C). The stationary phase was a Halo Fused C18 column (0.5 × 100 mm, 2.7 μm; Eksigent Technologies, Dublin, OH, USA). The mobile phase was a mixture of 0.1% (v/v) formic acid in water (A) and 0.1% (v/v) formic acid in ANC (B), and it was eluting at a flow-rate of 15.0 μL/min and at an increasing concentration of solvent B, that is, from 2% to 40% in 30 min. The injection volume was 4.0 μL. The oven temperature was set at 40 °C. The LC system was interfaced with a 5600+ Triple TOFTM system (AB Sciex, Concord, Canada), equipped with DuoSprayTM Ion Source and a calibrant delivery system. The mass spectrometer worked in data dependent acquisition mode (DDA). Peptide profiling was performed using a 100–1,300 Da mass range (TOF scan with an accumulation time of 100.0 ms), followed by an MS/MS product ion scan from 200 to 1,250 Da (accumulation time of 5.0 ms) with the abundance threshold set at 30 cps (35 candidate ions can be monitored per cycle). The ion source parameters were set in electrospray positive mode as follows: curtain gas (N2) at 25 psig, nebulizer gas GAS1 at 25 psig and GAS2 at 20 psig, ion spray floating voltage at 5,000 V, source temperature at 450 °C and declustering potential at 25 V (Cvijetic et al., 2017; Martinotti et al., 2016).

### Protein database search

The DDA files were searched using Mascot v. 2.4 (Matrix Science Inc., Boston, MA, USA). Trypsin was specified as a digestion enzyme with two missed cleavages. The instrument was set at ESI-QUAD-TOF, and the following modifications were allowed for the search: carbamidomethylcysteins as fixed modification and oxidized methionine as variable modification. A search tolerance of 50 ppm was specified for the peptide mass tolerance, and 0.1 Da for the MS/MS tolerance. The peptide charges searched for were set at 2^+^, 3^+^, and 4^+^, and the search was performed on monoisotopic mass. The unreviewed UniProt Swiss-Prot *B. mori* database (version 2017.06.21, containing 18320 sequence entries) was used. Only proteins with at least four peptides with a peptide score > peptide identity were considered for identification purposes. The mass spectrometry proteomics data have been deposited to the ProteomeXchange Consortium via the PRIDE partner repository with the dataset identifier PXD012869.

### Statistical analysis

Data from the nutrient profile analysis were compared by means of ANOVA and successively with the Tukey multiple comparison test, with the significance threshold set at *P* < 0.05.

The normalized spot intensity data were exported and analyzed using R statistic software (R version 3.3.2 - 2016-10-31). Data quality was assessed using distribution plots (frequency histograms), a box plot and the Shapiro Wilks normality test for all the considered proteins (Pedreschi et al., 2008). A control of the quality data was carried out, according to these preliminary data analyses, and the missing values were substituted with intra-spot medians when there were 3 or fewer missing values, or the intra-spots were erased and an experimental treatment was conducted in the case of 4 or 5 missing values; when the number of missing values was greater, the spot was eliminated from the statistical analysis. Data were analyzed by means of ANOVA, and the Tukey multiple comparison test was then used as a post hoc test for comparison of the means between treatments. Protein spots with a fold change ≥±1.5 and *P* < 0.05 were selected and excised from the gel for identification.

A multivariate analysis of the normalized spot quantities was performed using PAST software, version 2.17 (Hammer, Harper & Ryan, 2001). In order to further normalize the spot intensities, the quantitative data were standardized by subtraction of the mean spot values, and then dividing them by their standard deviations (*N* = 36). The standardized intensities were then ordered by means of a principal component analysis, in which each sample was labeled with differently shaped points. The same analysis was performed another time, but only on the significantly different spots, as selected by means of ANOVA and fold variation. Finally, the Manhattan algorithm was used to cluster the different samples according to the standardized intensities of those spots that showed a PCA correlation value >± 65%.

## Results

### Insect growth and nutrient composition

The mean weight, proximate composition, and energy value of the silkworm pupae are reported in Table 1. The insect growth was comparable for the two diets, in terms of the length of the cycle, albeit with a slight delay, ranging from 1 to 2 days, over the whole larval life span. The larvae reared on the A diet showed a slightly (but not significant) lower weight at the end of the fifth instar. The nutrient composition of the silkworm pupae grown on either the A or L diets was significantly different as far as its crude protein content and energy value are concerned. The highest protein content (16.8%) was recorded in female reared on artificial diet (F_A_), while the lowest (13.8%) was found in male reared on mulberry leaves diet (M_L_), as a result of the different protein contents (on a DM basis) of the diets (Table 1). The energy silkworm pupae results showed an opposite trend, with respect to the protein content, with the highest values recorded in both the M_L_ and F_L_, that is, 6.82 ± 0.79 and 6.02 ± 0.39 Mj/kg, respectively.

**Table 1:** Mean weight, proximate composition, and energy value of SWP.

	Experimental groups			
	M_A_	M_L_	F_A_	F_L_
Mean weight (g)	0.80 ± 0.05^b^	0.83 ± 0.16^b^	1.00 ± 0.13^a^	1.01 ± 0.20^a^
Dry matter (% FM)	23.40 ± 0.18	25.10 ± 1.47	23.26 ± 0.65	25.90 ± 1.39
Crude protein (% FM)	14.38 ± 1.63^ab^	13.81 ± 2.41^b^	16.83 ± 1.43^a^	14.58 ± 1.94^ab^
Ash (% FM)	1.33 ± 0.07	1.16 ± 0.17	1.27 ± 0.10	1.30 ± 0.29
Gross energy (Mj/kg FM)	5.38 ± 0.19^bc^	6.82 ± 0.79^a^	5.09 ± 0.21^c^	6.02 ± 0.39^b^

**Note:**

Values are means ± standard deviations of triplicate analyses; FM, fresh matter means with the different letters in the same row are significantly different (*P* < 0.05).

### Proteomic analysis: identification of differentially expressed proteins

**Figure 1:**
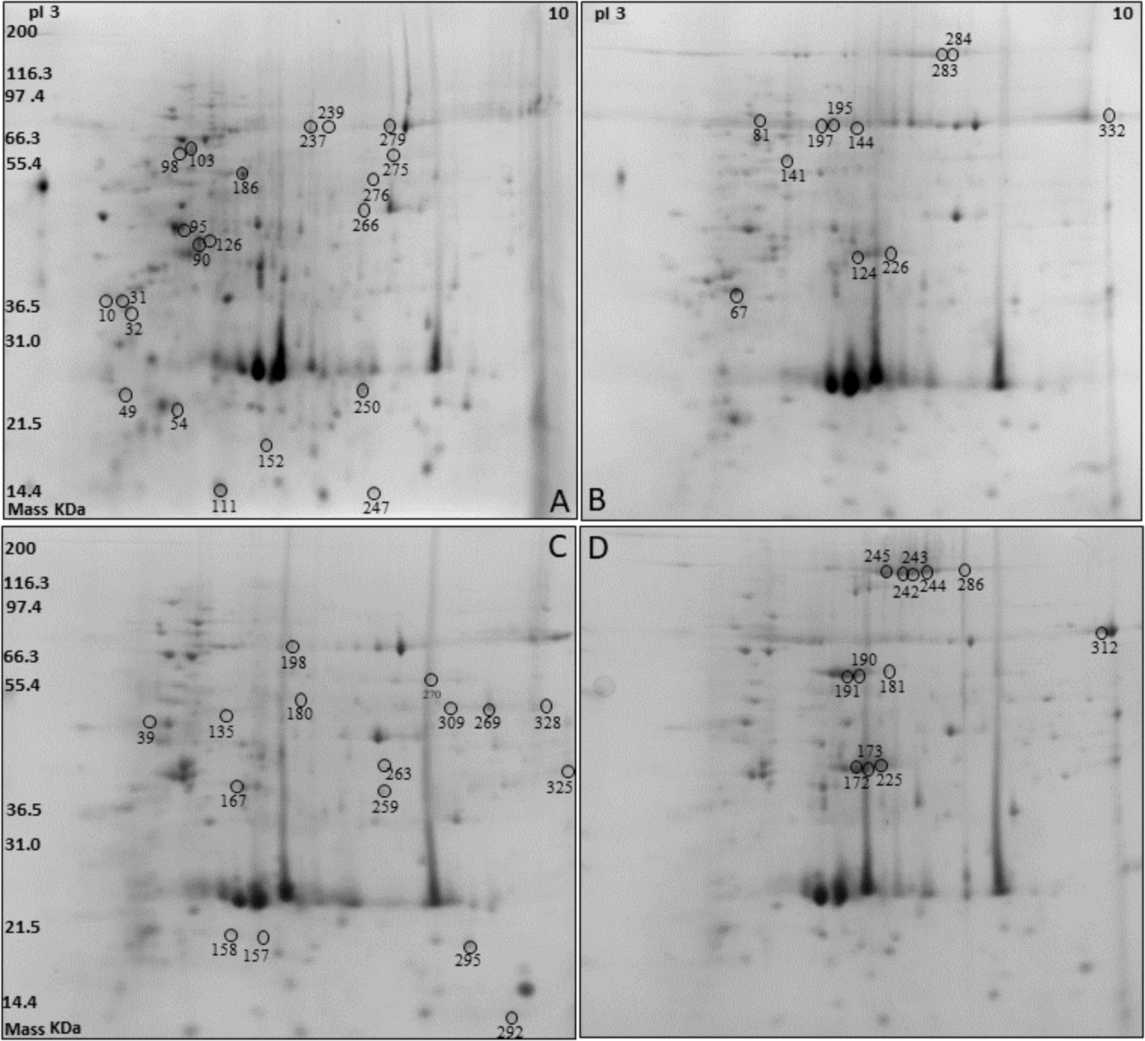
Two-dimensional electrophoresis (2DE) silkworm reared on two different diets considering male separated from female: Artificial diet (A, male; B, female) and fresh mulberry leaves diet (C, male; D, female).

Two-dimensional electrophoresis was performed on protein extracts from silkworm pupae reared on two different diets (A and L diets) and considering males (M) separately from females (F) (Fig. 1). Overall, 153 ± 9, 158 ± 15 spots were detected in the M and F reared on the A diet, while 157 ± 21, 163 ± 26 spots were detected in the M and F reared on the L diet. The protein spots that resulted to be differentially expressed (*P* < 0.05) with a fold change ≥± 1.5, under different sex and diet conditions, were selected and excised from the gel for their identification by means of mass spectrometry analysis.

Following the mass spectrometry analyses, the proteins listed in Table 2 were chosen as the best candidates obtained by bioinformatics search, with at least four valid peptides (Table S2). Some proteins and/or isoforms were identified in more than one spot on the same gel: Vitellogenin (spots 124, 172, 173, 226 as Vitellogenin light chain; spots 242, 243, 244, 245, 283, 284, 286 as Vitellogenin heavy chain, and spot 225), Catalase (spots 269, 276, and 309), Chitinase (spots 98 and 103), Transferrin (spots 239 and 279) and Egg specific protein (spots 190 and 191). In some cases, two or more proteins were identified in one spot: for instance, sex specific storage protein 1, sex specific storage protein 2, and arylphorin were identified in spots 144, 195, and 197. However, it was not possible to discriminate which of these proteins was responsible for the spot volume variation.

**Table 2:** Summary of differentially expressed protein identified by LC-MS.

Spot	Entry UniProt	Name	Mass_t_/Mass_e_	pI_t_/pI_e_	Peptides	Protein score	Coverage (%)
10	C0H6F9	Putative cuticle protein	28,335/36,000	4.63/4.30	8	545	49.6
31	C0H6F9	Putative cuticle protein	28,335/36,000	4.63/4.50	11	1,733	59.6
32	B9VTR5	32 kDa apolipoprotein	32,299/34,000	4.79/4.60	11	1,625	51.2
39	Q8T8B2	Tubulin beta chain	50,638/52,000	4.75/4.80	15	1,414	33.8
	Q8I9N4	Masquerade-like serine proteinase homolog	46,764/52,000	4,96/4,80	15	633	40
49	B9VTR5	32 kDa apolipoprotein	32,299/22,000	4.79/4.90	8	1,981	27.4
54	Q8T113	27 kDa glycoprotein	25,571/23,000	5.12/5.10	9	1,387	56.4
67	Q03383	Antichymotrypsin-1	44,715/40,000	5.21/5.00	12	458	29.5
81	H9JP12	Sex-specific storage-protein 1	88,007/82,000	5.28/5.00	20	689	22.5
90	H9IXK0	Antichymotrypsin-1	41,893/45,000	5.14/5.20	27	2,417	60.2
95	C4PAW6	Hemolin	45,335/50,000	5.12/5.20	32	4,911	80.2
98	Q9GQC4	Chitinase	61,886/65,000	5.01/5.20	20	1,270	41
100	I6XKQ0	Heat shock protein 70-5	75,536/80,000	5.84/5.70	13	623	22.5
	H9IXK0	Heat shock cognate protein	71,359/80,000	5.33/5.70	9	354	17.4
103	Q8WR52	Chitinase	64,280/65,000	5.14/5.20	15	576	31.4
111	Q2QEH2	Cellular retinoic acid binding protein	14,963/65,000	5.66/5.20	16	1,760	76.5
124	Q27309	Vitellogenin	203,725/40,000	6.85/6.00	16	1,087	10.5
126	H9IXK0	Antichymotrypsin-1	41,893/45,000	5.14/5.10	29	2,062	57.6
135	Q2F5Y9	Mitochondrial aldehyde dehydrogenase	53,127/54,000	5.57/5.70	14	696	31.1
141	P49010	Chitooligosaccharidolytic beta-*N*-acetylglucosaminidase	68,968/60,000	5.17/5.70	24	1,871	39.3
	H9J8Q7	Beta-hexosaminidase	61,914/60,000	5.33/5.30	24	1,836	44.5
144	Q1HPP4	Arylphorin	83,569/80,000	5.7/6.00	37	1,925	53.9
	H9JP12	Sex-specific storage-protein 1	88,007/80,000	6.78/6.00	35	2,164	43.9
	P20613	Sex-specific storage-protein 2	83,698/80,000	6.04/6.00	34	1,785	48.4
152	Q1HPP5	Actin-depolymerizing factor 1	17,227/18,000	6.17/6.00	17	1,219	81.8
157	Q5CCJ4	Glutathione S-transferase sigma	23,382/23,000	5.85/6.20	17	1,237	71.1
158	Q5CCJ4	Glutathione S-transferase sigma	23,382/23,000	5.85/5.80	16	1,177	67.2
167	Q2F5T5	Arginine kinase	40,308/40,000	5.87/5.90	24	1,841	60.6
172	Q27309	Vitellogenin (light chain)	40,203/40,000	6.85/6.30	22	3,021	65.6
173	Q27309	Vitellogenin (light chain)	40,203/40,000	6.85/6.30	23	2,861	71.3
180	H9J859	Fascin	57,239/55,000	6.25/6.50	16	967	39.3
181	H9JLS3	Dynein heavy chain 2, axonemal-like	386,433/60,000	6.42/6.50	20	759	6
186	H9JGR2	Chitinase precursor	61,037/58,000	5.58/5.90	36	4,098	66.4
190	Q17219	Egg-specific protein	63,545/60,000	6.14/6.30	30	3,745	71.2
191	Q17219	Egg-specific protein	63,545/60,000	6.14/6.20	38	4,668	78.5
195	Q1HPP4	Arylphorin	83,569/80,000	5.70/6.50	55	3,186	74.1
	P09179	Sex-specific storage-protein 1	87,890/80,000	6.78/6.50	57	5,231	67.3
	P20613	Sex-specific storage-protein 2	83,698/80,000	6.04/6.50	43	2,619	58.1
197	Q1HPP4	Arylphorin	83,569/80,000	5.70/6.50	55	3,186	74.1
	P09179	Sex-specific storage-protein 1	87,890/80,000	6.78/6.50	57	5,231	67.3
	P20613	Sex-specific storage-protein 2	83,698/80,000	6.04/6.50	43	2,619	58.1
198	H9JTA2	Uncharacterized protein	74,049/73,000	6.31/6.40	16	498	30.5
	Q27451	Phenoloxidase subunit 1	79,305/73,000	6.25/6.40	16	402	26.0
225	Q27309	Vitellogenin	203,725/40,000	6.85/6.50	21	2,198	17.6
226	Q27309	Vitellogenin (light chain)	40,203/40,000	6.85/6.80	18	1,779	62.3
237	H9JP12	Sex-specific storage-protein 1	88,007/80,000	6.78/6.80	31	1,728	44.1
239	O97158	Transferrin	77,156/80,000	6.89/7.00	21	1,032	39.4
242	Q27309	Vitellogenin (heavy chain)	161,327/160,000	6.85/6.80	36	2,062	26.8
243	Q27309	Vitellogenin (heavy chain)	161,327/160,000	6.85/7.00	35	1,921	24.1
244	Q27309	Vitellogenin (heavy chain)	161,327/160,000	6.85/7.10	37	1,835	27.1
245	Q27309	Vitellogenin (heavy chain)	161,327/160,000	6.85/6.80	40	2,323	30.7
247	Q1HPS1	ML-domain containing secreted protein	17,360/17,000	6.28/7.20	5	324	27.9
250	Q1HQ02	Ferritin	26,245/25,000	6.75/7.00	12	843	49.8
259	Q1HPN7	Fructose-bisphosphate aldolase	39,971/40,000	8.38/7.70	15	1,376	46.4
263	H9ITY5	Probable medium-chain specific acyl-CoA dehydrogenase. mitochondrial isoform X2	46,461/45,000	5.91/7.50	16	856	44.1
266	A7BEX9	Imaginal disk growth factor	48,362/50,000	7.64/7.20	19	1,734	52.8
269	Q68AP5	Catalase	57,092/55,000	8.11/8.20	34	2,149	67.5
270	H9IYX7	Bifunctional purine biosynthesis protein	64,577/60,000	7.19/7.80	31	1,977	49.6
275	H9IYX7	Bifunctional purine biosynthesis protein	64,577/62,000	7.19/7.50	25	1,193	49.2
276	H9IZ23	Pyruvate kinase	68,697/55,000	9.00/7.20	16	941	27.8
	Q68AP5	Catalase	57,092/55,000	8.11/7.20	10	362	22
	H9J8X4	Glucose-6-phosphate 1-dehydrogenase	56,942/55,000	6.86/7.20	11	288	28
279	O97158	Transferrin	77,156/80,000	6.89/7.50	50	4,089	71.8
283	Q27309	Vitellogenin (heavy chain)	161,327/160,000	6.85/7.30	57	3,470	47.7
284	Q27309	Vitellogenin (heavy chain)	161,327/160,000	6.85/7.40	65	4,638	51.4
286	Q27309	Vitellogenin (heavy chain)	161,327/160,000	6.85/7.50	50	3,175	40.6
292	Q69FX2	Promoting protein	17,625/16,000	8.37/8.80	10	863	68.8
295	Q60GK5	Glutathione S-transferase delta	24,269/23,000	7.61/8.30	20	2,143	91.2
309	Q68AP5	Catalase	57,092/55,000	8.11/8.00	25	1,430	58.6
312	G1UIS8	Apolipophorin protein	371,420/73,000	7.94/9.00	24	1,511	7.8
325	C6L8Q2	Putative acetyl transferase	41,580/40,000	8.91/9.30	16	1,082	59.3
	A0A0A0QY84	Elongation factor 1-alpha	50,626/40,000	9.24/9.30	17	1,013	41.7
328	Q2F5T3	ATP synthase subunit alpha	59,792/55,000	9.21/9.00	26	1,447	51.2
332	H9JP12	Sex-specific storage-protein 1	88,007/80,000	6.78/9.30	17	679	20.02
	Q1HPP4	Arylphorin	83,569/80,000	5.70/9.30	18	476	26.3

Among the diet-modulated spots, confirmed for both genders, seven were only detected in the silkworms reared on the A diet (spots 10, 32, 54, 67, 98, 103, and 141), nine were only detected in the silkworms reared on the L diet (spots 157, 180, 181, 198, 269, 270, 292, 325, and 328), 10 spots were up-regulated in the silkworms reared on the A diet (spots 49, 124, 144, 186, 237, 244, 266, 279, 283, and 284) and only one spot was up-regulated in the silkworms reared on the L diet (spot 167) (Table 3).

**Table 3:** List of diet-related protein; protein with loading value greater than 0.65 are bold type.

Protein	Spot *N*	Statistically significant RATIO	Fold-change	Loading value
Spots detected only in the silkworm reared on A diet comparing the same gender				
Putative cuticle protein	10	M_A_/M_L_	Only in M_A_	0.359
		F_A_/F_L_	Only in F_A_	
	31	F_A_/F_L_	Only in F_A_	0.391
**32 kDa apolipoprotein**	32	M_A_/M_L_	Only in M_A_	**0.677**
		F_A_/F_L_	Only in F_A_	
27 kDa glycoprotein	54	M_A_/M_L_	Only in M_A_	0.580
		F_A_/F_L_	Only in F_A_	
Antichymotrypsin-1	67	M_A_/M_L_	Only in M_A_	0.086
		F_A_/F_L_	Only in F_A_	
	90	M_A_/M_L_	Only in M_A_	−0.224
Hemolin	95	F_A_/F_L_	Only in F_A_	0.292
Chitinase	98	M_A_/M_L_	Only in M_A_	0.556
		F_A_/F_L_	Only in F_A_	
	103	M_A_/M_L_	Only in M_A_	0.614
		F_A_/F_L_	Only in F_A_	
Heat shock protein 70-5	100	M_A_/M_L_	Only in M_A_	−0.055
Heat shock cognate protein				
Antichymotrypsin	126	M_A_/M_L_	Only in M_A_	0.320
Beta-hexosaminidase	141	M_A_/M_L_	Only in M_A_	0.515
Acetylglucosamidase		F_A_/F_L_	Only in F_A_	
Actin-depolymerizing1	152	M_A_/M_L_	Only in M_A_	0.214
Glutathione S-transferase sigma	158	F_A_/F_L_	Only in F_A_	0.379
Transferrin	239	M_A_/M_L_	Only in M_A_	0.312
ML-domain containing secreted protein	247	M_A_/M_L_	Only in M_A_	0.167
Imaginal disk growth factor	266	F_A_/F_L_	Only in F_A_	0.495
SP1	332	F_A_/F_L_	Only in F_A_	0.227
Arylphorin				
Spots detected only in the silkworm reared on L diet comparing the same gender				
Tubulin beta chain	39	M_L_/F_L_	Only in M_L_	−0.493
Masquerade-like serine proteinase homolog				
SP1	81	F_A_/F_L_	Only in F_L_	0.223
Mitochondrial aldehyde dehydrogenase	135	F_A_/F_L_	Only in F_L_	−0.448
Glutathione S-transferase sigma	157	M_A_/M_L_	Only in M_L_	−0.428
		F_A_/F_L_	Only in F_L_	
**Fascin**	180	M_A_/M_L_	Only in M_L_	**−0.717**
		F_A_/F_L_	Only in F_L_	
Dynein heavy chain 2	181	M_A_/M_L_	Only in M_L_	−0.508
		F_A_/F_L_	Only in F_L_	
Egg-specific protein	190	F_A_/F_L_	Only in F_L_	−0.039
	191	F_A_/F_L_	Only in F_L_	0.103
SP1	195	M_A_/M_L_	Only in M_L_	0.101
SP2	197	M_A_/M_L_	Only in M_L_	0.209
Arylphorin				
Phenoloxidase subunit 1	198	M_A_/M_L_	Only in M_L_	−0.214
		F_A_/F_L_	Only in F_L_	
Spots detected only in the silkworm reared on L diet comparing the same gender				
SP1	237	F_A_/F_L_	Only in F_L_	0.222
**acyl-CoA dehydrogenase isoform X2**	263	F_A_/F_L_	Only in F_L_	**−0.734**
**Catalase**	269	F_A_/F_L_	Only in F_L_	**−0.783**
		M_A_/M_L_	Only in M_L_	
	309	M_A_/M_L_	Only in M_L_	**−0.745**
**Bifunctional purine biosynthesis protein**	270	M_A_/M_L_	Only in M_L_	**−0.779**
		F_A_/F_L_	Only in F_L_	
Promoting protein	292	M_A_/M_L_	Only in M_L_	−0.422
		F_A_/F_L_	Only in F_L_	
Spots detected only in the silkworm reared on L diet comparing the same gender				
Apolipophorin protein	312	F_A_/F_L_	Only in F_L_	−0.208
Putative acetyl transferase	325	M_A_/M_L_	Only in M_L_	−0.619
Elongation factor 1-alpha		F_A_/F_L_	Only in F_L_	
ATP synthase α subunit	328	M_A_/M_L_	Only in M_L_	−0.636
		F_A_/F_L_	Only in F_L_	
Spots up-regulated in the silkworm reared on A diet				
32 kDa-apolipoprotein	49	M_A_/M_L_	2.35*	0.460
Vitellogenin LC	124	F_A_/F_L_	2.25*	0.209
SP1	144	F_A_/F_L_	2.94*	0.499
SP2				
Arylphorin				
Chitinase precursor	186	M_A_/M_L_	2.29*	0.574
SP1	237	M_A_/M_L_	2.16*	0.222
Imaginal disk growth factor	266	M_A_/M_L_	2.17**	0.495
Transferrin	279	M_A_/M_L_	2.64**	0.606
Vitellogenin HC	244	F_A_/F_L_	1.78**	0.460
	283		1.71*	0.419
	284		3.55*	0.437
Spots up-regulated in the silkworm reared on L diet				
Arginine kinase	167	M_L_/M_A_	1.88*	0.046

**Notes:**

F_A_, female artificial diet; F_L_, female mulberry leaves; M_A_, male artificial diet; M_L_, male mulberry leaves.

* *P* < 0.05.

** *P* < 0.01.

Considering the sex-modulated spots confirmed for both diets, three were only detected in M (spots 167, 275, and 276), eight were only detected in F (spots 124, 172, 173, 225, 242, 244, 283, and 284), 11 were up-regulated in M (spots 10,32, 95, 11, 135, 157, 198, 250, 259, 295, and 328) and two were up-regulated in F (spots 67 and 181) (Table 4). In addition, 15 spots were only present in one condition: spots 100, 126, 152, 239, and 247 were only detected in M_A_, spots 39 and 309 were only detected in M_L_, spots 81 and 332 were only detected in F_A_ and spots 190, 191, 243, 245, 286, and 312 were only detected in F_L_.

**Table 4:** List of sex-related protein; protein with loading value greater than 0.65 are bold typed.

Protein	Spot *N*	Statistically significant RATIO	Fold-change	Loading value
Spots detected only in Male comparing silkworm reared on the same diet				
Putative cuticle protein	31	M_L_/F_L_	Only in M_L_	0.218
Tubulin beta chain	39	M_L_/F_L_	Only in M_L_	0.064
Masquerade-like serine proteinase homolog				
Hemolin	95	M_L_/F_L_	Only in M_L_	0.544
Heat shock protein 70-5	100	M_A_/F_A_	Only in M_A_	0.571
Heat shock cognate protein				
Antichymotrypsin	126	M_A_/F_A_	Only in M_A_	0.553
Mitochondrial aldehyde dehydrogenase	135	M_A_/F_A_	Only in M_A_	0.497
Actin-depolymerizing factor 1	152	M_A_/F_A_	Only in M_A_	0.474
Arginine kinase	167	M_A_/F_A_	Only in MA	0.219
		M_L_/F_L_	Only in ML	
SP1	237	M_A_/F_A_	Only in M_A_	−0.159
Transferrin	239	M_A_/F_A_	Only in M_A_	−0.138
ML-domain containing secreted protein	247	M_A_/F_A_	Only in M_A_	0.336
acyl-CoA dh isoform X2	263	M_A_/F_A_	Only in MA	0.257
Imaginal disk growth factor	266	M_L_/F_L_	Only in ML	**0.674**
Bifunctional purine biosynthesis protein	275	M_A_/F_A_	Only in M_A_	0.545
		M_L_/F_L_	Only in ML	
**Pyruvate kinase**	276	M_A_/F_A_	Only in M_A_	**0.716**
**Catalase**				
**Glucose-6P-1 dehydrogenase**		M_L_/F_L_	Only in ML	
Transferrin	279	MA/FA	Only in MA	0.534
Spots detected only in Female comparing silkworm reared on the same diet				
SP1	81	M_A_/F_A_	Only in F_A_	−0.422
Antichymotrypsin-1	90	M_L_/F_L_	Only in F_L_	0.492
**Egg-specific protein**	190	M_L_/F_L_	Only in F_L_	−0.531
	191	M_L_/F_L_	Only in F_L_	**−0.681**
**Vitellogenin LC**	124	M_A_/F_A_	Only in F_A_	−0.536
		M_L_/F_L_	Only in F_L_	
	172	M_A_/F_A_	Only in F_A_	−0.575
		M_L_/F_L_	Only in F_L_	
	173	M_A_/F_A_	Only in F_A_	**−0.674**
		M_L_/F_L_	Only in F_L_	
	225	M_A_/F_A_	Only in F_A_	**−0.663**
		M_L_/F_L_	Only in F_L_	
	226	M_A_/F_A_	Only in F_A_	−0.437
Vitellogenin HC	242	M_A_/F_A_	Only in F_A_	−0.631
		M_L_/F_L_	Only in F_L_	
	243	M_L_/F_L_	Only in F_L_	−0.619
	244	M_A_/F_A_	Only in F_A_	−0.571
		M_L_/F_L_	Only in F_L_	
	245	M_L_/F_L_	Only in F_L_	−0.591
	283	M_A_/F_A_	Only in F_A_	−0.494
		M_L_/F_L_	Only in F_L_	
	284	M_A_/F_A_	Only in F_A_	−0.437
		M_L_/F_L_	Only in F_L_	
	286	M_L_/F_L_	Only in F_L_	−0.529
Apolipophorin protein	312	M_L_/F_L_	Only in F_L_	−0.255
SP1	332	M_A_/F_A_	only in F_A_	−0.544
Arylphorin				
Spots up-regulated in Male				
Putative cuticle protein	10	M_A_/F_A_	2.96**	0.633
**32 kDa apolipoprotein**	32	M_A_/F_A_	1.70**	**0.677**
Hemolin	95	M_A_/F_A_	2.34*	0.544
**Cellular retinoic acid binding protein**	111	M_A_/F_A_	3.23**	**0.729**
Mitochondrial aldehyde dehydrogenase	135	M_L_/F_L_	1.87*	0.497
Glutathione S-transferase sigma	157	M_L_/F_L_	2.63**	0.100
Phenoloxidase subunit 1	198	M_L_/F_L_	1.72	−0.081
**Ferritin**	250	M_A_/F_A_	2.31**	**0.706**
**Fructose-bisphosphate aldolase**	259	M_A_/F_A_	1.71*	**0.767**
		M_L_/F_L_	2.04*	
Glutathione S-transferase delta	295	M_L_/F_L_	2.23*	0.474
ATP synthase α subunit	328	M_L_/F_L_	1.75**	0.051
Spots up-regulated in Female				
Antichymotrypsin-1	67	F_A_/M_A_	1.73**	0.011
Dynein heavy chain 2	181	F_L_/M_L_	1.84**	−0.593

**Notes:**

F_A_, female artificial diet; F_L_, female mulberry leaves; M_A_, male artificial diet; M_L_, male mulberry leaves.

* *P* < 0.05.

** *P* < 0.01.

**Figure 2:**
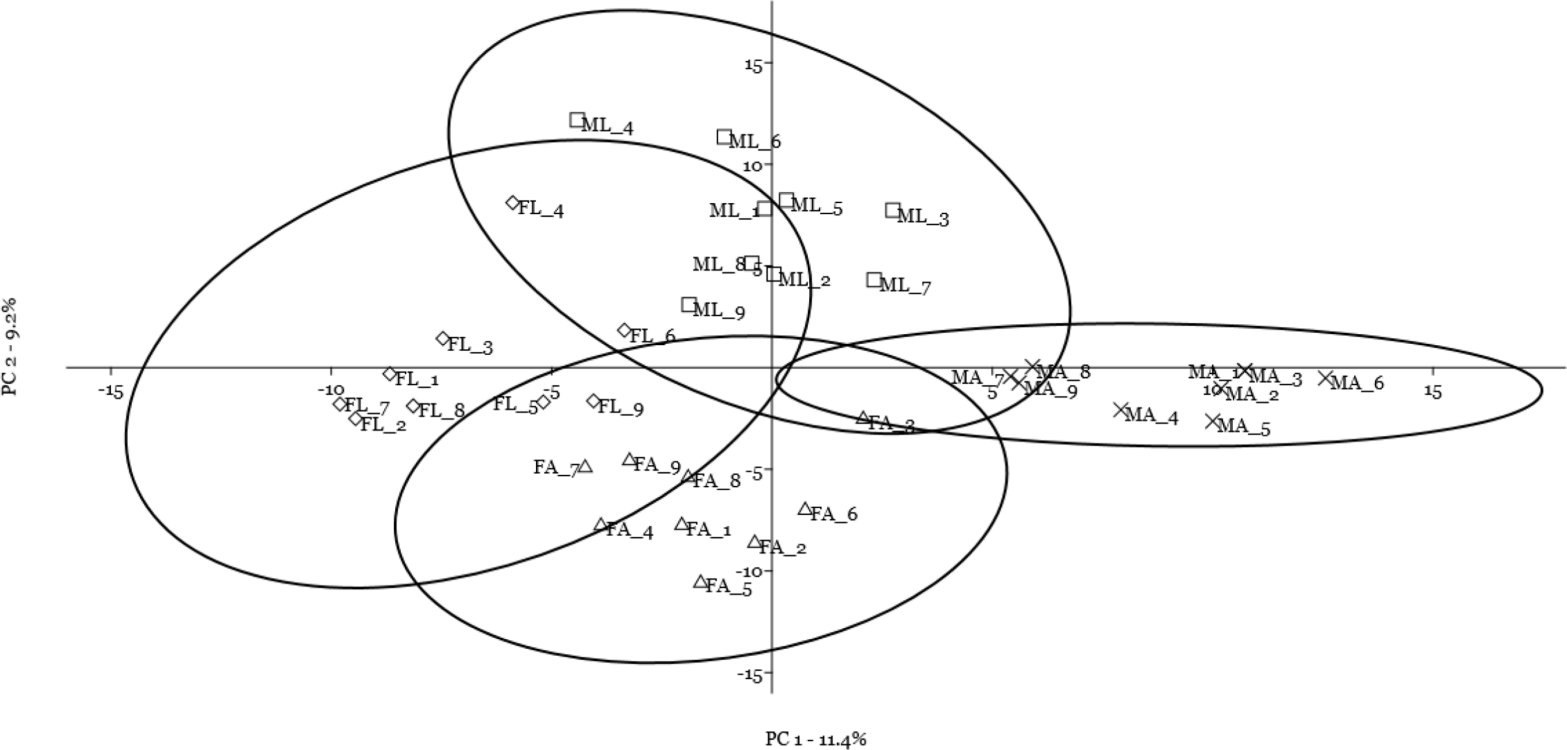
Principal component analysis plot of all quantitative data from two-dimensional electrophoresis gel image analysis. Female silkworm reared on artificial diet (F_A_ empty triangle) or on mulberry leaves (F_L_ empty diamond); Male silkworm reared on artificial diet (M_A_ black cross) or on mulberry leaves (M_L_ empty square).

**Figure 3:**
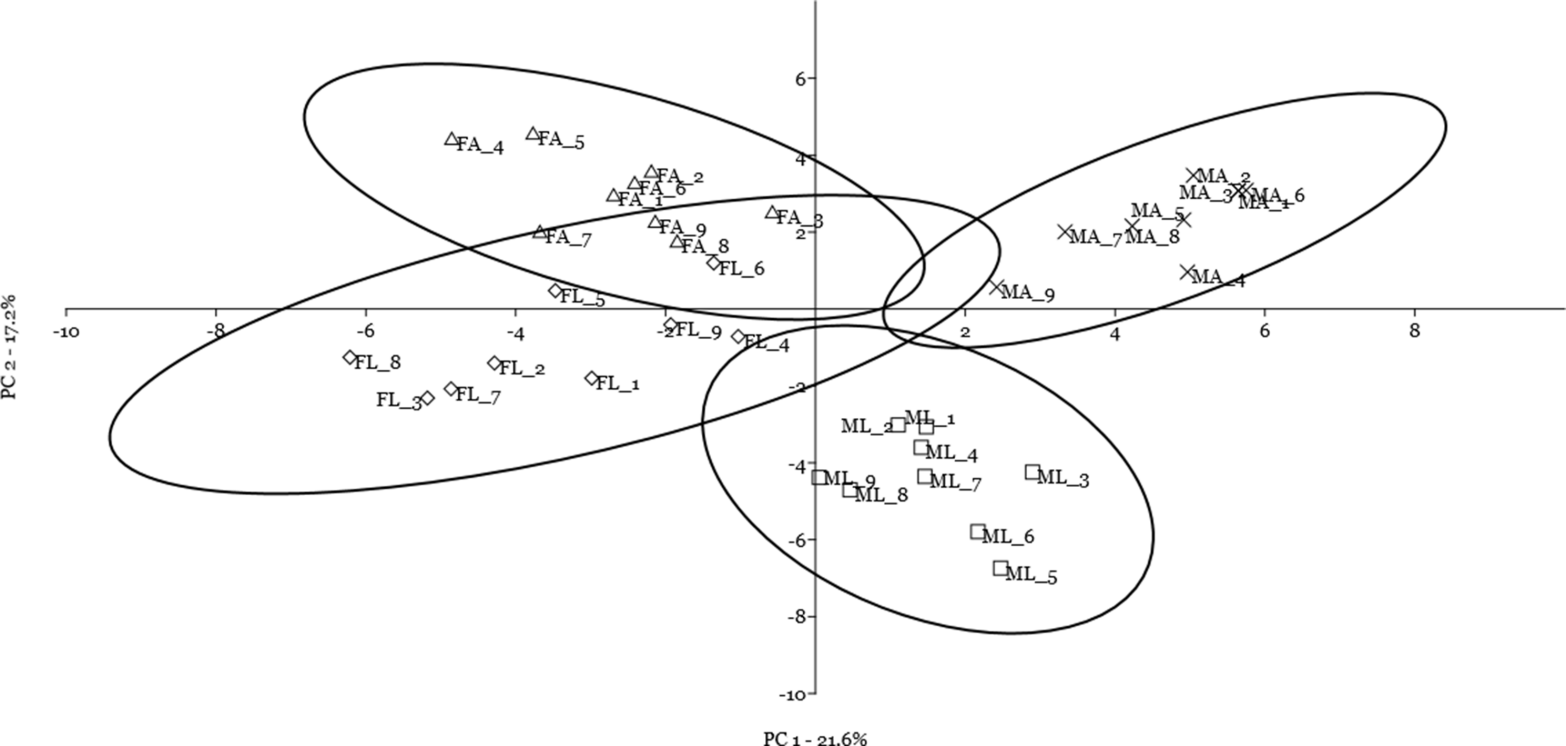
Principal component analysis plot of the spots that were significantly different according to univariate statistics and up/downregulated more than 1.5-fold. Female silkworm reared on artificial diet (F_A_ empty triangle) or on mulberry leaves (F_L_ empty diamond); Male silkworm reared on artificial diet (M_A_ black cross) or on mulberry leaves (M_L_ empty square).

A multivariate statistical approach (PCA) was used, in two steps, to investigate the clustering tendencies and to outline the contribution of single spots to the differences between samples. Figure 2 reports the grouping tendency of the four samples when all standardized quantitative data from 2DE gel image analysis were included. The different samples seemed to cluster quite separately according to their spot intensity, with the sex disclosed along the second principal component, and the diet separated, although less sharply, along the first principal component. The PCA was then repeated by including only quantitative data of the spots that were significantly different according to univariate statistics and up/downregulated more than 1.5-fold (Fig. 3). Again in this case, the four samples showed a tendency to group, according to sex, along the first principal component, and to diet along the second component. By analyzing the contribution of the single spots to these components (loading values in Tables 2 and 3), we identified the spots that were the most relevant for the variability between groups.

**Figure 4:**
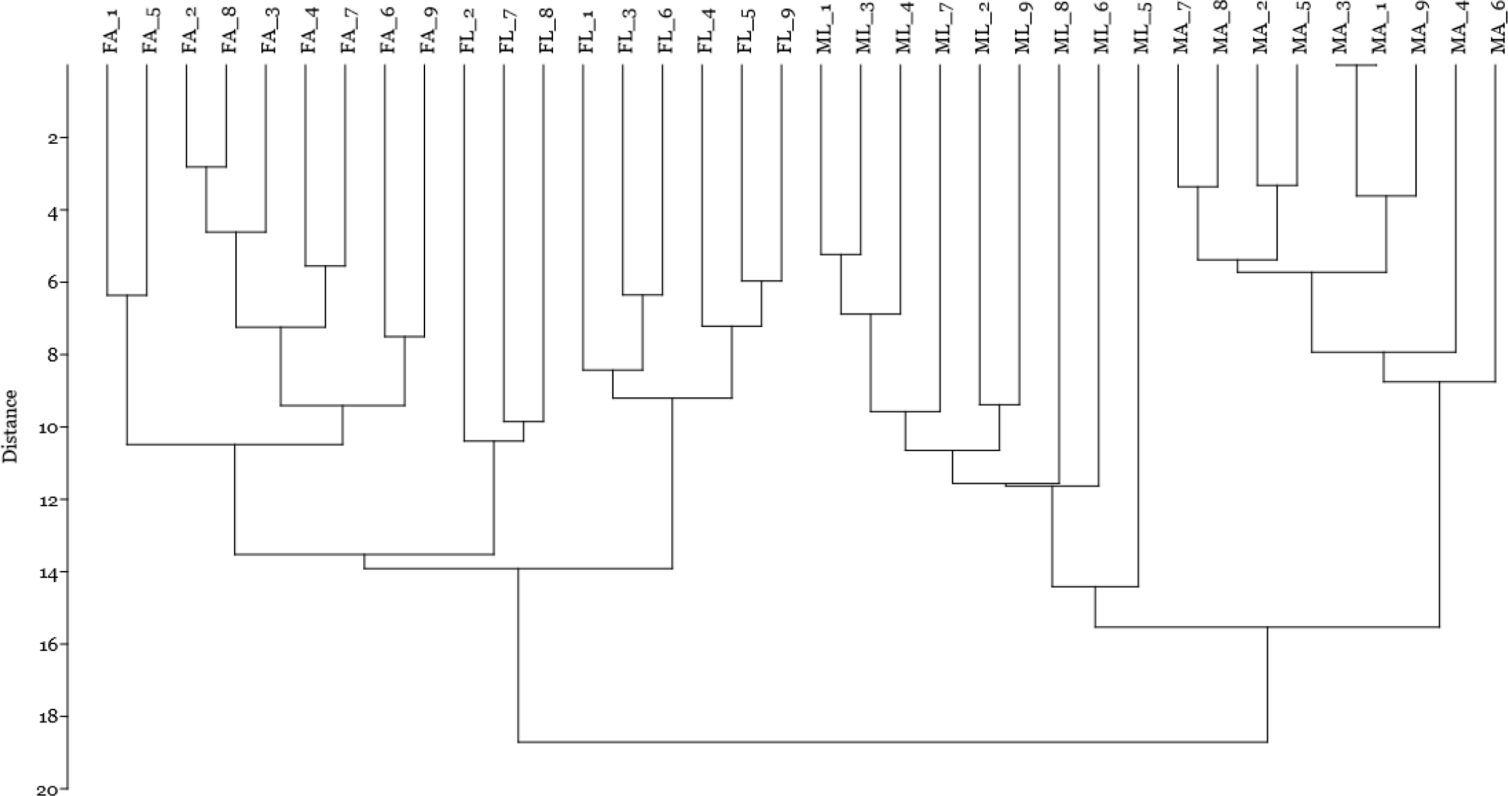
Cluster analysis by Manhattan algorithm of quantitative data on spot volume for those 14 spots with loading value greater than 0.65. Female silkworm reared on artificial diet (F_A_) or on mulberry leaves (F_L_); Male silkworm reared on artificial diet (M_A_) or on mulberry leaves (M_L_).

Finally, by including the quantitative data on spot volume for the 14 selected spots in a cluster analysis using the Manhattan algorithm (Fig. 4), we observed that these proteins were suitable for discriminating sex and diet effects as separate clusters, with a higher sex- than diet-related effect.

## Discussion

### Insect growth and nutrient composition

It is worth noting that the insect growth results of the silkworm pupae reared on mulberry leaves are referred to good quality leaves produced in springtime; however, if the quality of the leaves had not been optimal (e.g., late summer leaves), the differences might not have been significant, or the reverse situation might have been obtained, where a lighter cocoon weight would have been obtained for the leaves rather than for the diet (Kumar et al., 2013).

Regardless of which rearing substrate was utilized, the protein content of the silkworm pupae was found to be higher than that of other data reported for silkworm pupae that are produced as by-products of reeling industry (Rao, 1994; Pereira et al., 2003). The lower caloric value of the silkworm pupae reared on the A diet could be related to its lower cholesterol content, as previously reported by Dong et al. (2017) in a metabolomics study in which a significantly lower cholesterol content was found in both F (62%) and M (71.4%) reared on A diet compared to L diet.

### Protein discriminating gender effect and protein discriminating diet effect

By using the multivariate statistical approach, seven proteins were found to better discriminate the sex effect, whereas five proteins were better at discriminating the diet effect. Egg-specific protein (Q17219) and vitellogenin (Q27309) were only present in females. The imaginal disk growth factor (IDGF, A7BEX9), cellular retinoic acid (RA) binding protein (CRABP, Q2QEH2), ferritin (Q1HQ02), fructose-bisphosphate aldolase (Q1HPN7), and 32 kDa apolipoprotein (B9VTR5) were up-regulated in males. The bifunctional purine biosynthesis protein (H9IYX7), acyl-CoA dehydrogenase (H9ITY5), Fascin (H9J859), and Catalase (Q68AP5) were up-regulated in the L diet; while 32 kDa apolipoprotein (B9VTR5) were up-regulated in the A diet.

Vitellogenin (Vg) is the major precursor of the egg-yolk protein Vitellin, together with the egg-specific protein are the major proteins in yolk. In our experiments, both Vg and the egg-specific proteins were only detected in the female silkworms, as expected.

The Vg protein in *B. mori* (BmVg), is a tetramer with a molecular mass of 440 kDa, composed of two heavy chains and two light chains (Izumi, Tomino & Chino, 1980). In our 2DE experiments, we found Vg in 12 spots, separated at different pI (from 6.5 to 7.5) and molecular weight (40 kDa for the light chain and 160 kDa for the heavy chain). Vg was found in the female pupae without any differences between the L and A diets, thus making Vg the best discriminating gender-protein.

The egg-specific protein showed a correlation with gender, but, unlike Vg, it was only found in females reared on the L diet. Since the synthesis of egg-specific protein is stimulated by ecdysone (Ono, Nagayama & Shimura, 1975), its absence from the silkworm pupae reared on the A diet could be correlated with this hormone-dependent regulation, thus suggesting a hormonal balance alteration between the L and A diets. In previous data obtained for the same rearing conditions (Cappellozza et al., 2005), the silkworm pupae fed on an A diet often enclosed into moths that laid non-diapausing eggs, while they were usually monovoltine when reared on an L diet. This silkworm pupae behavior, which is mainly linked to hormone secretion, has already been demonstrated by Yamamura et al. (2011).

Moving on to the proteins that are more abundant in males, IDGF, showed up regulation in both of the diets, with more abundance in the A diet. IDGF is the first polypeptide growth factor to be reported for invertebrates, and it cooperates with insulin to stimulate the proliferation, polarization, and motility of imaginal disc cells (Hipfner & Cohen, 1999). IDGF has been suggested to be a systemic regulator in response to environmental inputs, such as nutritional status: the amount of BmIDGF dropped significantly after starvation and increased again upon re-feeding (Wang et al., 2009). A proteomic analysis on *B. mori* performed by Zhou et al. (2008) gave analogous results to ours, showing that the concentration of BmIDGF in the hemolymph was double in the larvae reared on A diet compared to those reared on L diet. CRABP is an exclusively sex-regulated protein belonging to the RA signal transduction pathway. RA is a vitamin A metabolite, that is, involved in the proliferation, cellular differentiation, remodeling of adult tissues, and in apoptosis, through the modulation of target gene expression. CRABP protects the *B. mori* cells from RA excesses, by sequestering RA and inducing its degradation (Wang et al., 2007). In our experiments, CRABP was over expressed in the males, for both of the diets, thus supporting the idea that RA, involved in several biological processes in females, have to be more suitable for females than for males.

Two other proteins were more up-regulated in the males than in the females: Fructose-bisphosphate aldolase (glycolytic pathway), and bifunctional purine biosynthesis protein. The bifunctional purine biosynthesis protein was found to be regulated in a similar way by Qin et al. (2014), who compared midgut proteins from *B. mori* male and female larvae. The authors demonstrated an enhancement in pyrimidine and purine biosynthesis in silkworm males. This up regulation may result in improved DNA/RNA synthesis and metabolism, which subsequently allow the male larvae to grow faster than the female ones in the fifth instar. In our experiment, a faster growth of the male larvae than the female ones was observed, as the cocoon emergence distributed over 3 days was recorded earlier for the male moths. This general behavior of anticipated emergence of male silk moths is well-known and it has been explored carefully to synchronize males and females for mating in the egg production process of silkworms for commercial purposes (Wang, 1989).

Moreover, the bifunctional purine biosynthesis protein, together with acetyl-CoA dehydrogenase were also upregulated (or found to be exclusively present) in the *B. mori* reared on the L diet. These results are in agreement with those of Dong, Pan & Zhang (2018), who demonstrated, by means of a metabolite analysis of *B. mori*, that both the carbohydrate and purine metabolisms were slowed down in silkworms reared on an A diet. Another diet-related protein was Fascin only biosynthesized in the silkworms reared on mulberry leaves, at the same extent between males and females. This is a globular actin cross-linking protein that bundles actin filaments into organized structures (Cant et al., 1994). A functional study on sea urchin demonstrated the importance of Fascin in the organization of F-actin in the egg microvillus core, which forms shortly after fertilization (Otto, Kane & Bryan, 1982). Zhou et al. (2008) showed a decreased expression of Tropomyosin 1 in *B. mori* reared on an A diet compared to an L diet: they speculated that the down-regulation of tropomiosin might inhibit the formation of actin filaments, therefore, weakening the contraction ability of the smooth muscle in the midgut of silkworms. In our experiments, Fascin, which is involved in the same biological process as Tropomyosin, showed the same expression profile, and it might, therefore, also be responsible for the reduction of actin structure organization in *B. mori* reared on A diets. Catalase, just like Fascin, was only present in the silkworms reared on the L diet. This is the protein mainly considered to be responsible for the scavenging of the reactive oxygen species (ROS) (Sohal, Arnold & Orr, 1990). ROS are produced as a consequence of aerobic respiration and substrate oxidation and are responsible for the damage of DNA, proteins, and lipid membranes. The cells biosynthesize antioxidative enzymes, such as Catalase, to protect themselves from ROS. Yamamoto et al. (2005) were the first to sequence and characterize *B. mori* Catalase (BmCAT), and some years later Nabizadeh & Kumar (2010) demonstrated a significant decrease in BmCAT activity in silkworms reared under thermal stress (at 40 ± 1 °C). In our experiments, BmCAT was absent in the silkworms reared on the A diet. Considering that, BmCAT in silkworm pupae is also linked to voltinism of the eggs, Zhao & Shi (2009) observed that the CAT activity in univoltine strains of *B. mori* was higher from the fifth to the seventh day of pupal development than that of polyvoltine strains. Therefore, this behavior might be linked to a variation in the hormonal balance rather than to a physiological disorder.

### Potentially allergenic proteins

The proteomic approach setup adopted in this study allowed us to verify whether the expression of the already known allergenic proteins in *B. mori* were differentially affected by sex and rearing substrates. Among the differentially regulated proteins identified in this study, we found three proteins that have already been demonstrated to be allergens in *B. mori*: arginine kinase (AK; Liu et al., 2009), 27 kDa glycoprotein (Jeong et al., 2016), and chitinase (Zhao et al., 2015).

Arginine kinases are enzymes involved in energy catabolism and are found exclusively in invertebrates. Several AKs have recently been characterized as allergens and they have subsequently been proposed to be panallergens (García-Orozco et al., 2007; Sookrung et al., 2006). Using sequence alignment analysis, Liu et al. (2009) determined that *Bm*AK shows significant similarity (ranging from 81% to 92%) with other AKs that have been associated with allergenicity. Moreover, they demonstrated that *Bm*AK reacts with sera from patients who have shown a reaction to the crude extract of silkworms during a skin prick test, and that cross-reacts with the AK from the *Periplaneta americana*, rPaAK cockroach.

The 27 kDa glycoprotein is synthesized in the fat body of silkwarm and it is present at all stages of development in both sexes. However, its function is still unknown. A 27-kDa hemolymph protein from the wax moth, *Galleria mellonella*, has been reported to be an inhalant allergen in a patient suffering from rhinoconjunctivitis (Madero et al., 2007), and it shares a 54.9% amino acid sequence identity with the 27-kDa glycoprotein of silkworms. This report suggests the possibility of a different sensitization route for the 27 kDa hemolymph allergen in insects. In the study of Jeong et al. (2016), a 27-kDa glycoprotein was identified from a silkworm pupa as a heat stable IgE binding component. Specific IgE to recombinant 27-kDa glycoprotein was detected for one third of the tested silkworm allergic subjects, and IgE reactivity was shown to be increased after the protein extract was heated, so Jeong et al. (2016) suggested that food processing might increase allergenicity of the 27-kDa glycoprotein as a result of chemical modifications and/or structural changes.

The main function of insect Chitinases pertains to the turnover of such chitin-containing extracellular matrices as the insect cuticle and the peritrophic matrix during molting. In addition, chitinases may have a digestive function in insects, if their diet contains chitin. Zhao et al. (2015) found that silkworm chitinase resembles the Der f 18 of *Dermatophagoides farinae* (Q86R84) (24.8% of identical amino acid and 57.4% similar). They investigated IgE reactivity to *Bm*Chitinase using sera of patients allergic to silkworm pupa protein, and speculated that silkworm chitinase might be a cross-reactive allergen of house dust mites (Der f 18). Further studies are needed to identify the specific epitopes of these potentially allergenic proteins.

In our experiment, AK (spot 167) resulted to be upregulated in the males for both of the diets. 27-kDa glycoprotein (spot 54) was upregulated in the A diet while Chitinase (spots 98 and 103) was only present in the silkworms reared on the A diet and was upregulated in the males. From an allergenic point of view, our data indicate that female silkworms reared on mulberry leaves contain lower levels of known allergens, compared to the other experimental conditions that were considered. Further studies to assess the safety of *B. mori*, from the allergenic point of view, if used as food or a food ingredient, including the use of the sera of patients allergic to insect/crustaceous/dust mite are necessary.

## Conclusions

A comparative proteomic experiment has been conducted to investigate the difference in the *B. mori* pupa protein profile, as affected by diet and gender. A PCA analysis allowed to outline the contribution of single proteins to differences in the experimental conditions: seven and five pupa proteins were found to be more effective in discriminating the sex and the diet type, respectively. Overall, we found that the pupae derived from silkworms grown on artificial diets and mulberry leaves show differences in their protein composition, although these differences did not lead to any different physiological traits. On the other hand, the differential protein expression between the two diets has highlighted a general flexibility of the insect to adapt to the artificial diet. Larvae developed on the two alternative feeding substrates show important differences in proteins related to lipid transport and metabolism; this phenomenon might be responsible for the recorded variation in silk production and, through the egg composition, might have an influence on the progeny physiological behavior.

Although this is a preliminary study, it has been possible to claim that female silkworm pupae reared on mulberry leaves contain lower levels of known allergens than those reared in the other experimental conditions. However, these results need to be supported by further immunoblotting experiments with the sera of potentially allergic patients.

The present work can provide some basic understanding of *B. mori* growth and physiology in relation to gender and farming. In addition, the data presented here offer a contribution to the evaluation of the influence of these two factors on the allergen profile of *B. mori* for its use as food or as a food ingredient.

## Supplemental Information

10.7717/peerj.6723/supp-1 Supplemental Information 1 Supplemental information: Composition of the artificial diet.

10.7717/peerj.6723/supp-2 Supplemental Information 2 List of identified proteins with at least 4 valid peptides.

## Additional Information and Declarations

### Data Availability

The following information was supplied regarding data availability: The mass spectrometry proteomics data have been deposited to the ProteomeXchange Consortium via the PRIDE partner repository with the dataset identifier PXD012869.
