# Supplementary material for: Investigation of the protein profile of silkworm (Bombyx mori) pupae reared on a well-calibrated artificial diet compared to mulberry leaf diet
Source: PeerJ. 2019 Jun 12;7:e6723. doi: 10.7717/peerj.6723 (PMC6571003; doi:10.7717/peerj.6723)
Supplement: Supplemental Information 1 [file peerj-07-6723-s001.docx]

**Table S1**. Composition of the artificial diet^a^

| Ingredient | Quantities/100 g dry weight^b^ |
| --- | --- |
| Defatted soybean meal | 36.0 g |
| Dried mulberry leaf powder | 25.0 g |
| Soybean fiber | 5.0 g |
| Agar | 4.2 g |
| Corn starch | 4.0 g |
| Citric acid | 4.0 g |
| Salt mixture | 3.0 g |
| Ascorbic acid | 2.0 g |
| Propionic acid | 691.0 mg |
| *β*-sitosterol | 500.0 mg |
| Vitamin mixture | 399.0 mg |
| Sorbic acid | 200.0 mg |
| Chloramphenicol | 10.0 mg |
| a Adapted from Cappellozza et al. (2005)  b The powder was hydrated in the ratio of 1 g dry powder: 2.6 g of water. | |
