# Supplementary material for: Investigation of the protein profile of silkworm (Bombyx mori) pupae reared on a well-calibrated artificial diet compared to mulberry leaf diet
Source: PeerJ. 2019 Jun 12;7:e6723. doi: 10.7717/peerj.6723 (PMC6571003; doi:10.7717/peerj.6723)
Supplement: Supplemental Information 2 [file peerj-07-6723-s002.docx]

| **Spot** | **Entry UniProt** | **Name** | **MW_t_/MW_e_** | **pI_t_/pI_e_** | **Coverage** | **Protein score** | **Peptide sequence** |
| --- | --- | --- | --- | --- | --- | --- | --- |
| **10** | C0H6F9 | Putative cuticle protein | 28335/36000 | 4.63/4.3 | 49.6% | 545 | WQALDALK  TVADVEASAK  AADLAQAAADK  SIATQPPVEEVK  TAEAQLDGAVASQAVQLAK  AVVNTNLAQEQAVDGVWAVEDKK  SAVGAAPYVVAPVFPVVYPGIASPAIK  TSAVESDAQTSGVLGAGHISTIQGAIATK |
| **31** | C0H6F9 | Putative cuticle protein | 28335/36000 | 4.63/4.5 | 59.6% | 1733 | WQALDALK  TVADVEASAK  AADLAQAAADK  KWQALDALK  SIATQPPVEEVK  VEGNTDSVAVEAK  AIAINEQNAENYNVK  TAEAQLDGAVASQAVQLAK  AVVNTNLAQEQAVDGVWAVEDK  SAVGAAPYVVAPVFPVVYPGIASPAIK  TSAVESDAQTSGVLGAGHISTIQGAIATK |
| **32** | B9VTR5 | 32 kDa apolipoprotein | 32299/34000 | 4.79/4.6 | 51.2% | 1625 | FFTVSELGSR  QIIDEVVQER  LMEGSLQMIGR  FLGTWYEAER  EKEPVEDSDSVK  CVTTNYVSTPEGR  EKEPVEDSDSVKK  IIVSNEIVNSLTGMKR  ERLPSLMAMQNAYAVLDR  TNQADCTILPDPVAIPIEAK  SAVPEISFEPKPVPVPEMILTENEK |
| **39** | Q8T8B2 | Tubulin beta chain | 50638/52000 | 4.75/4.8 | 33.8% | 1414 | YLTVAAIFR  FPGQLNADLR  LAVNMVPFPR  ISEQFTAMFR  FPGQLNADLRK  KLAVNMVPFPR  INVYYNEASGGK  IMNTYSVVPSPK  EVDEQMLNIQNK  AILVDLEPGTMDSVR  LHFFMPGFAPLTSR  LHFFMPGFAPLTSR  ALTVPELTQQMFDAK  NSSYFVEWIPNNVK  EIVHIQAGQCGNQIGAK |
|  | Q8I9N4 | Masquerade-like serine proteinase homolog | 46764/52000 | 4,96/4,8 | 40% | 633 | ERAPAGVR  NPDGVAFR  VDVPVVDR  CFATGWGK  NTCQSQLR  TTGDVDGETK  KVDVPVVDR  EIYPYQDR  TWIDDKVAGK  AGEWDTQNTK  NTCQSQLRR  EIVIHKDFNK  FGEFPWMVAILK  VEPVDDNEPEGQK  GDGGSPLVCPIDYEK |
| **49** | B9VTR5 | 32 kDa apolipoprotein | 32299/22000 | 4.79/4.9 | 27.4% | 1981 | FFTVSELGSR  LMEGSLQMIGR  FLGTWYEAER  RLMEGSLQMIGR  CVTTNYVSTPEGR  IIVSNEIVNSLTGMK  LPSLMAMQNAYAVLDR  ERLPSLMAMQNAYAVLDR |
| **54** | Q8T113 | 27 kDa glycoprotein | 25571/23000 | 5.12/5.1 | 56.4% | 1387 | TCFLNLK  GLVDLNVLK  DSPCHTALPK  VDEMTSCIVK  NNAEDKVPEVEAALR  IALFIAEGGPECFQQK  QSFPTVESANNLSLVEK  TEIEEAKPNGALDEVFKK  SLEECSTPTPANMAESLIK |
| **67** | Q03383 | Antichymotrypsin-1 | 44715/40000 | 5.21/5 | 29.5% | 458 | LADAFSR  NAADIINR  NDQVVVSR  AFQEAFVK  DIELEIPK  IYVSDQYK  SGLDLNTILPK  SEVDNINFSAPK  GLLEDVFNLSPAGR  MIELPYKEPGFR  DIELEIPKFEIR  MVVVLPDKIDGLPAVLEK |
| **81** | H9JP12 | Sex-specific storage-protein 1 | 88007/82000 | 5.28/5 | 22.5% | 689 | EGILTGK  TVDAVVR  DFDVFMR  DLGMSNTSK  DLGMSNTSK  FADVMIYR  EQFSFPGVK  SEDIENLAR  TSDMTFMAR  VIHLTNLMK  EPMVNLDMK  VIHLTNLMK  EPMVNLDMK  LPSGDEMPVR  VCNIFTVFK  IRLPSGDEMPVR  LVLGGLEIVGDDAK  SCDKYMNVDVVK  RGEIMMYANQQLLAR  SSMDMQGFIPEYLSTR |
| **90** | H9IXK0 | Antichymotrypsin-1 | 41893/45000 | 5.14/5.2 | 60.2% | 2417 | LGDAIDK  TPVSEDK  LADAFSR  FLTVANK  YAEFDPK  NAADIINR  DFHVDEK  MIELPYK  MVVVLPDK  NDQVVVSR  AFQEAFVK  DIELEIPK  TRLGDAIDK  ILFAGTHTH  IYVSDQYK  ESYNLADDK  IDGLPAVLEK  LGDAIDKTSLK  IFQEPAPGIVK  WADEQTQGHIK  GLLEDVFNLSPAGR  MIELPYKEPGFR  DIELEIPKFEIR  VDHPFLFLILHEDK  LLQSLFYTENEELGAK  MVVVLPDKIDGLPAVLEK  IFQEPAPGIVKNDQVVVSR  EILGGGEAQESSHTYGLLNQR  VDHPFLFLILHEDKILFAGTHTH |
| **95** | C4PAW6 | Hemolin | 45335/50000 | 5.12/5.2 | 80.2% | 4911 | SDFGVASTR  EAPAEVLFR  DGVNVDNTYK  TYIETPAFEEK  VTVVEGKPFELR  DGVNVDNTYKDR  ASDEGEYQCFAK  TYIETPAFEEKK  RTYIETPAFEEK  KVTVVEGKPFELR  TLATQGEDVTIPCK  RTYIETPAFEEKK  RTLATQGEDVTIPCK  HHDEDGSTENFMDR  HHDEDGSTENFMDRR  CPVPGGYPKPTISWMR  LECATEGDDSGVEYSWR  LECATEGDDSGVEYSWRK  EVWAEDAGTYTCDVDNQAGR  LQHTITFSVVSAPTFTTKPEK  EVWAEDAGTYTCDVDNQAGRR  RLQHTITFSVVSAPTFTTKPEK  LQHTITFSVVSAPTFTTKPEKR  VGDLTYLYCIYGGTPLAHPSWSK  LVIKEVWAEDAGTYTCDVDNQAGR  DGMHFSVGQDTLTTIDAGSLVFTQTK  SNQGYYGCTASNEHGAEYAETALQVA  LVIKEVWAEDAGTYTCDVDNQAGRR  KDGMHFSVGQDTLTTIDAGSLVFTQTK  ATGIPSPLVSWTYNGEPVTEGVTGDGLVIK  LVCMASSPAADEGVPIVTYYITQVTPASEPTYGELIPQYLSDHVVAK |
| **98** | Q9GQC4 | Chitinase | 61886/65000 | 5.01/5.2 | 41% | 1270 | SVVDFLK  KWDEFGK  SVVDFLKK  YSHMVAQK  LVVGIPFYGR  GLCGEENPLIK  HMSTYTVPPAR  GTQWVGYEDPR  RPHDQWAYEK  FMVAVGGWAEGGSK  LNVNDGLNLWEEK  GWELTAAVPLANFR  GNWAGFADVHSPLYK  SSEVLIIDPELDVDK  ADRGWELTAAVPLANFR  SFTLSAGNNNYGLGTYINK  IVCYFSNWAVYRPGVGR  GGSFSDKDEFLYFVQELK  KYDFDGLDLDWEYPGAADR  GGSFSDKDEFLYFVQELKR |
| **100** | I6XKQ0 | Heat shock protein 70-5 | 75536/80000 | 5.84/5.7 | 22.5% | 623 | NTTIPTK  DQGIDIR  LFEQAYK  AAEQFAAADK  ALQDAEVQR  DAGQISGLNVLR  TATGQLQQASLK  VQQTVQEIFGR  SKLESLVGDLIR  SDIGEVLLVGGMTR  VYSPSQIGAFVLIK  MKETAEAYLNTSVK  VEVANQAEAVLHDTDTK |
|  | H9IXK0 | Heat shock cognate protein | 71359/80000 | 5,33/5,7 | 17.4% | 354 | NTTIPTK  LLQDFFNGK  DAGTISGLNVLR  VEIIANDQGNR  AQIHDIVLVGGSTR  ARFEELNADLFR  TTPSYVAFTDTER  IINEPTAAAIAYGLDKK  TVQNAVITVPAYFNDSQR |
| **103** | Q8WR52 | Chitinase | 64280/65000 | 5.14/5.2 | 31.4% | 576 | SVVDFLK  KWDEFGK  LVVGIPFYGR  FLYFVQELK  GLCGEENPLIK  HMSTYTVPPAR  RPHDQWAYEK  FLYFVQELKR  LNVNDGLNLWEEK  GWELTAAVPLANFR  GNWAGFADVHSPLYK  SSEVLIIDPELDVDK  SFTLSAGNNNYGLGTYINK  IVCYFSNWAVYRPGVGR  KYDFDGLDLDWEYPGAADR |
| **111** | Q2QEH2 | Cellular retinoic acid binding protein | 14963/65000 | 5.66/5.2 | 76.5% | 1760 | TIGVGLITR  EFGPEEMK  TIGVGLITRK  APDGLEVTYVR  AANAVTPTVELR  SVCTFEGNTLK  AANAVTPTVELRK  KAANAVTPTVELR  FKPGEEFEEDR  MTSSENFDEFMK  KAANAVTPTVELRK  DGDEYNLVTSSTFK  QVQKAPDGLEVTYVR  KDGDEYNLVTSSTFK  YKMTSSENFDEFMK  FKPGEEFEEDRADGAK |
| **124** | Q27309 | Vitellogenin | 203725/40000 | 6.85/6 | 10.5% | 1087 | ELPVDLK  EQQQGLFR  TQEQQLIGR  YQPTPNIDK  VFEIEIDGGR  VIHDSQNNYDR  FANEQDPVEVTK  GLISALQLDTSAHR  TLAHLQEGPSSGSAFK  AETTSTVHVHPHLYGK  LENPQHGNFNEQLPDPR  VAYHFGVPVGAEWTGTAHK  IVSLDFPTSVPVPQENLIK  YQPTPNIDKVFEIEIDGGR  FSSQSQSTGGQTYPSPWQVGK  VIHDSQNNYDREQQQGLFR |
| **126** | H9IXK0 | Antichymotrypsin-1 | 41893/45000 | 5.14/5.1 | 57.6% | 2062 | LGDAIDK  TPVSEDK  LADAFSR  FLTVANK  YAEFDPK  NAADIINR  MVVVLPDK  MIELPYK  NDQVVVSR  AFQEAFVK  DIELEIPK  TRLGDAIDK  ILFAGTHTH  IYVSDQYK  ESYNLADDK  IDGLPAVLEK  LGDAIDKTSLK  IFQEPAPGIVK  WADEQTQGHIK  GLLEDVFNLSPAGR  MIELPYKEPGFR  DIELEIPKFEIR  LLQSLFYTENEELGAK  MVVVLPDKIDGLPAVLEK  IFQEPAPGIVKNDQVVVSR  EILGGGEAQESSHTYGLLNQR  EEIREILGGGEAQESSHTYGLLNQR |
| **135** | Q2F5Y9 | Mitochondrial aldehyde dehydrogenase | 53127/54000 | 5.57/5.7 | 31.1% | 696 | VLNYIVAGK  ADIDLAVAAAK  LAELLESQSR  TYVQSGIYDK  FETFDEVVDR  IILGAASAVNLKR  EEIFGPVQSILK  SPLVVFNDADVEK  LFINNEWVDAVSK  ELGEDGISQYLETK  LFINNEWVDAVSKK  IAREEIFGPVQSILK  YLAELETLDCGKPVK  ILGNTIPSDGEVLTFTMK |
| **141** | P49010 | Chitooligosaccharidolytic beta-N-acetylglucosaminidase | 68968/60000 | 5.17/5.7 | 39.3% | 1871 | AAAFAER  LGAYSPTK  NNLLIVR  GILLDTAR  GNSFFGVR  TLVSSSVPK  TIDAMAAVK  WTCENNR  LWNYFQK  DRPVYPYR  NFYSIDSIK  EPVLSLEACK  SDDLLTAAADR  WNLDKSSFLK  NFYSIDSIKR  INMNTIDIQITK  SDDLLTAAADRFK  VYGNSPAVMALSYR  MWAEPSTAWQDAEHR  NEPENKEPVLSLEACK  IRNEPENKEPVLSLEACK  RLPLILWTSTLTDYTHVEK  LNTFHWHITDSQSFPLVLQK  VLPEFDAPAHVGEGWQDTGLTVCFK |
|  | H9J8Q7 | Beta-hexosaminidase | 61914/60000 | 5.33/5.3 | 44.5% | 1836 | AAAFAER  LGAYSPTK  NNLLIVR  GILLDTAR  GNSFFGVR  TLVSSSVPK  TIDAMAAVK  LWNYFQK  DRPVYPYR  NFYSIDSIK  SDDLLTAAADR  WNLDKSSFLK  NFYSIDSIKR  INMNTIDIQITK  SDDLLTAAADRFK  VYGNSPAVMALSYR  SVTVYLVNDNPYIR  MWAEPSTAWQDAEHR  HGLETLSQLIVYDDIR  LPLILWTSTLTDYTHVEK  RLPLILWTSTLTDYTHVEK  LNTFHWHITDSQSFPLVLQK  VLPEFDAPAHVGEGWQDTGLTVCFK  WTCENNR |
| **144** | Q1HPP4 | Arylphorin | 83569/80000 | 5.7/6 | 53.9% | 1925 | LTNGLGK  LTAGQNK  AVEEFLK  LYFDGVK  SYEVFAR  IDFHDPK  VPNMYFK  FLDTYEK  ITDVKVDK  DFETFYK  TFVQFLQK  NVDAVFVEK  ENDYFVYK  DHFEAFGQK  NSNEFVIFK  IVEYIVEFK  VLGAAPMPFDK  TGYYPLMLTK  NLEFSVFYDK  DIFIYHEGER  EDSVPMTEIMK  QYLKPYTQDK  DPAFYQLYNR  RVLGAAPMPFDK  MFMNMEVLQK  FFELDWFTTK  IPEFSWYSPIK  FNIPSYDTQSNVVPK  DIFIYHEGERFPYK  DYDIEMNMDNYTNKK  YDDNGIPLTLEDNWMK  SDCHGFVVPAPYEVYPK  IGKDYDIEMNMDNYTNKK  ILSFFQDVSQLNTDDEYYK  NSNEFVIFKEDSVPMTEIMK  AINFVGNYWQDNADLYGEEVTK  FTPFAQRPDYYNLHTEENYER |
|  | H9JP12 | Sex-specific storage-protein 1 | 88007/80000 | 6.78/6 | 43.9% | 2164 | EGILTGK  TVDAVVR  LMSVNDK  DGTVISLK  DFDVFMR  DGTVISLKK  YMNVDVVK  DLGMSNTSK  QFMEMYK  QNNMVVATK  AAKDPVLWK  FADVMIYR  EQFSFPGVK  SEDIENLAR  TSDMTFMAR  VIHLTNLMK  LPSGDEMPVR  EPMVNLDMK  VCNIFTVFK  FADVMIYRK  DPVFWMIMK  KSEDIENLAR  IRLPSGDEMPVR  MLSYGQYNMDK  EQFSFPGVKVEK  LVLGGLEIVGDDAK  QMMDDVEMMIR  LDMFELDSFMYK  RLDMFELDSFMYK  RGEIMMYANQQLLAR  LLDHILQPTMFEDIK  LNHHPFQVSIDVMSDK  SSMDMQGFIPEYLSTR  GETFVHTNELQMEEAVK  LLDHILQPTMFEDIKEIAK |
|  | P20613 | Sex-specific storage-protein 2 | 83698/80000 | 6.04/6 | 48.4% | 1785 | LTNGLGK  LTAGQNK  AVEEFLK  LYFDGVK  SYEVFAR  IDFHDPK  FLDTYEK  ITDVKVDK  DFETFYK  TFVQFLQK  NVDAVFVEK  ENDYFVYK  DHFEAFGQK  NSNEFVIFK  IVEYIVEFK  VLGAAPMPFDK  TGYYPLMLTK  SKNVDAVFVEK  NLEFSVFYDK  MFMNMEVLQK  EDSVPMTEIMK  QYLKPYTQDK  DPAFYQLYNR  RVLGAAPMPFDK  FFELDWFTTK  IPEFSWYSPIK  MQHGLINPEAAAK  DYDIEMNMDNYTNKK  YDDNGIPLTLEDNWMK  IGKDYDIEMNMDNYTNKK  ILSFFQDVSQLNTDDEYYK  NSNEFVIFKEDSVPMTEIMK  AINFVGNYWQDNADLYGEEVTK  FTPFAQRPDYYNLHTEENYER |
| **152** | Q1HPP5 | Actin-depolymerizing factor 1 | 17227/18000 | 6.17/6 | 81.8% | 1219 | SLVGVQK  GGTGECR  KSLVGVQK  TTYEEIK  YVVFYIR  TTYEEIKK  QIDVETVGER  MLYSSSFDALK  YVVFYIRDEK  MLYSSSFDALKK  LFLMSWCPDTAK  KMLYSSSFDALKK  NAEYEQFLEDLQK  YIQATDLSEASQEAVEEK  YIQATDLSEASQEAVEEKLR  YGLFDFEYTHQCQGTSEASK  YGLFDFEYTHQCQGTSEASKK |
| **157** | Q5CCJ4 | Glutathione S-transferase sigma | 23382/23000 | 5.85/6.2 | 71.1% | 1237 | ALGESQR  LNEILTK  AYVDAAPR  NNGHIALGK  FYYFPVK  YPFFFEK  ASAASVHYEK  KPIEAVLAIPK  QYAQSTAICR  AMLQKPDLEQK  ISSENWPEFKPK  TPFGQMPVLEIDGK  ASAASVHYEKDEAVK  LLLAYGGQEFEDNR  AELEETKYPFFFEK  KAELEETKYPFFFEK  AMLQKPDLEQKYPAFR |
| **158** | Q5CCJ4 | Glutathione S-transferase sigma | 23382/23000 | 5.85/5.8 | 67.2% | 1177 | ALGESQR  LNEILTK  NNGHIALGK  FYYFPVK  YPFFFEK  ASAASVHYEK  KPIEAVLAIPK  QYAQSTAICR  AMLQKPDLEQK  ISSENWPEFKPK  TPFGQMPVLEIDGK  ASAASVHYEKDEAVK  LLLAYGGQEFEDNR  AELEETKYPFFFEK  KAELEETKYPFFFEK  AMLQKPDLEQKYPAFR |
| **167** | Q2F5T5 | Arginine kinase | 40308/40000 | 5.87/5.9 | 60.6% | 1841 | LQGSDSK  LEAGFSK  LEEVASK  EVFDSLK  KLEEVASK  GIYHNENK  FLQAANACR  LVSAVNEIEK  MGLTEYDAVK  KIPFSHHDR  GTFYPLTGMSK  LVSAVNEIEKK  EMYDGIAELIK  VSGTLSSLEGELK  GEHTEAEGGVYDISNK  IISMQMGGDLQQVYK  TFLVWCNEEDHLR  ETQQQLIDDHFLFK  LGFLTFCPTNLGTTVR  IISMQMGGDLQQVYKR  EMEDKVSGTLSSLEGELK  GTRGEHTEAEGGVYDISNK  NWGDVDTLGNLDPAGEFVVSTR  MGLTEYDAVKEMYDGIAELIK |
| **172** | Q27309* | Vitellogenin  (light chain) | 40203/40000 | 6.85/6.3 | 65.6% | 3021 | ELPVDLK  EQQQGLFR  TQEQQLIGR  YQPTPNIDK  VFEIEIDGGR  STNYGHCHHR  VIHDSQNNYDR  FANEQDPVEVTK  GLISALQLDTSAHR  TLAHLQEGPSSGSAFK  AETTSTVHVHPHLYGK  ELPKFANEQDPVEVTK  LENPQHGNFNEQLPDPR  VAYHFGVPVGAEWTGTAHK  IVSLDFPTSVPVPQENLIK  YQPTPNIDKVFEIEIDGGR  FSSQSQSTGGQTYPSPWQVGK  VIHDSQNNYDREQQQGLFR  EGPIYKAETTSTVHVHPHLYGK  METDVTGDCETLYTVSPVASEWR  KMETDVTGDCETLYTVSPVASEWR  KMETDVTGDCETLYTVSPVASEWRR |
| **173** | Q27309* | Vitellogenin  (light chain) | 40203/40000 | 6.85/6.3 | 71.3% | 2861 | ELPVDLK  EQQQGLFR  TQEQQLIGR  YQPTPNIDK  VFEIEIDGGR  STNYGHCHHR  VIHDSQNNYDR  FANEQDPVEVTK  GLISALQLDTSAHR  TLAHLQEGPSSGSAFK  AETTSTVHVHPHLYGK  ELPKFANEQDPVEVTK  LENPQHGNFNEQLPDPR  VAYHFGVPVGAEWTGTAHK  IVSLDFPTSVPVPQENLIK  YQPTPNIDKVFEIEIDGGR  FSSQSQSTGGQTYPSPWQVGK  VIHDSQNNYDREQQQGLFR  METDVTGDCETLYTVSPVASEWR  AEVYSHVHMELISVDQDSGAEWPR  KMETDVTGDCETLYTVSPVASEWR  KMETDVTGDCETLYTVSPVASEWRR  QKAEVYSHVHMELISVDQDSGAEWPR |
| **180** | H9J859 | Fascin | 57239/55000 | 6.25/6.5 | 39.3% | 967 | YLNAGGK  INANGTSLK  CEQGFVGPK  GYFLGSSSDK  YALHTCNNK  YLTAETFGFK  ANYETIQVVR  DGSGAYLAPIGSK  GWWTIGLINSR  FHISVSDDNSGR  LADADLASATHWEY  GYFLGSSSDKLTCTAK  YYFYLINRPILVLK  DELFSLEDSLPQAAFVAALNDK  YLAVDSFGNVTCESEEKEPGSK  LQDTCTSECLFSAEYHAGALALR |
| **181** | H9JLS3 | Dynein heavy chain 2, axonemal-like | 386433/60000 | 6.42/6.5 | 6% | 759 | TPSTAPK  TLLSEVER  ESNLEAER  LAAFDLGLK  GSVLAYLSR  DLGNDCYK  NLNMEDER  LLESGLIALH  KGSVLAYLSR  ILQGMQQPSK  TVSLNPNWSK  EFDNAITHYEK  AENYEAALEDAEK  LEPTNQQLAQGLR  GNDALVNQNFDEAIK  ALELDPSNAEALEGYR  KGNDALVNQNFDEAIK  LAAFDLGLKDCEQCCK  AMSDPEVQQILRDPAMR  AYIDPVKAEQEKELGNEYFK |
| **186** | H9JGR2 | Chitinase precursor | 61037/58000 | 5.58/5.9 | 66.4% | 4098 | QANTGLK  IQVVNYK  DRFVASVK  DFLQTWK  GANPDLGSPK  LVVGVAMYGR  YLLTQGVQPK  GNFGQLMQLK  DGDVYVQLMK  GLSSWNEPYK  KLVVGVAMYGR  ETYTTDFGIR  EIEGSFQALQR  GTWQDGVVDYR  QMLDELAAETGR  EVWSGAGSATSAAFK  TYELTSAISAGWDK  FVASVKDFLQTWK  VVGAYFVEWGVYPR  YMDHIFVMSYDFK  VSIHDPWAALQKPQK  FFDGVDIDWEFPGGK  ELRQMLDELAAETGR  DRFVASVKDFLQTWK  EIVNGITSGTWQYFYDK  GANPDLGSPKDGDVYVQLMK  TFAIVEVNQAATAYNQLVTK  EAQKYMDHIFVMSYDFK  EDFKVSIHDPWAALQKPQK  VLPSIGGWTLADPFFFFTDK  GLSSWNEPYKGNFGQLMQLK  TFAIVEVNQAATAYNQLVTKK  TYELTSAISAGWDKIQVVNYK  VAQAPYVWNPTTGDLVTYDDAR  GWTGVHDYNDDIPFTGVANGPVK  VPVPNLTHLLYGFIPICGGDGINDSLK |
| **190** | Q17219 | Egg-specific protein | 63545/60000 | 6.14/6.3 | 71.2% | 3745 | MIAPTSK  IPPTHQTR  LQENLPNVK  YNYGAETNMK  TWDHFTDDAK  NTFHDAISETQR  QYGQNVASHDFR  EVDINHGDSVVWK  VYGASEPPSYDLSK  FYQYIHDQVGHGAFEPGK  SLAYMLSDAGYDVWLGNVR  VSAPVNLYHSHDAWLAHPK  QSFEVPEQQHFTDLDFQFSK  KKPVALLMHGLLGSADDWLLMGPSK  FSNDEIALHDLPAIIDHVLDISGQER  NIEMASGPNSPVQTEQDIEDIFGDSLK  APDTVYQK  KAPDTVYQK  HPALNDFWK  HHSPVYSVIMK  HLIETFGGAACR  TWDHFTDDAKK  DVEKLQENLPNVK  KNTFHDAISETQR  IVSMHALSPIVYMNYVR  QSFEVPEQQHFTDLDFQFSK  HQYPVEEHTVATDDGYHLTVLR  KHQYPVEEHTVATDDGYHLTVLR  LHYIGHSQGATTFFALMSEQPSYNEK  HVCNNLNYVISGINVYNQDADIVPVVMAHLPAGTSAR |
| **191** | Q17219 | Egg-specific protein | 63545/60000 | 6.14/6.2 | 78.5% | 4668 | APDTVYQK  HPALNDFWK  HHSPVYSVIMK  HLIETFGGAACR  TWDHFTDDAKK  NTFHDAISETQR  EVDINHGDSVVWK  DVEKLQENLPNVK  KNTFHDAISETQR  VMKQYGQNVASHDFR  SLAYMLSDAGYDVWLGNVR  HQYPVEEHTVATDDGYHLTVLR  KHQYPVEEHTVATDDGYHLTVLR  KKPVALLMHGLLGSADDWLLMGPSK  FSNDEIALHDLPAIIDHVLDISGQER  NIEMASGPNSPVQTEQDIEDIFGDSLK  LHYIGHSQGATTFFALMSEQPSYNEK  FYQYIHDQVGHGAFEPGKHLIETFGGAACR  HVCNNLNYVISGINVYNQDADIVPVVMAHLPAGTSAR  MIAPTSK  IPPTHQTR  KAPDTVYQK  LQENLPNVK  LMENMQNNS  YNYGAETNMK  TWDHFTDDAK  KYNYGAETNMK  QYGQNVASHDFR  VYGTSEPPSYDLSK  QYGQNVASHDFRK  SHVSKHPALNDFWK  IVSMHALSPIVYMNYVR  FYQYIHDQVGHGAFEPGK  VSAPVNLYHSHDAWLAHPK  QSFEVPEQQHFTDLDFQFSK  KPVALLMHGLLGSADDWLLMGPSK  VSAPVNLYHSHDAWLAHPKDVEK  QHSQDDIIQHPLDYVEQQIHQQK |
| **195** | Q1HPP4 | Arylphorin | 83569/80000 | 5.7/6.5 | 74.1% | 3186 | LTNGLGK  LTAGQNK  AVEEFLK  LYFDGVK  IDFHDPK  SYEVFAR  VPNMYFK  FLDTYEK  ITDVKVDK  DFETFYK  KAVEEFLK  TFVQFLQK  NVDAVFVEK  ENDYFVYK  DHFEAFGQK  NSNEFVIFK  IVEYIVEFK  VLGAAPMPFDK  TGYYPLMLTK  TKNVDAVFVEK  NLEFSVFYDK  DIFIYHEGER  EDSVPMTEIMK  QYLKPYTQDK  DPAFYQLYNR  RVLGAAPMPFDK  MFMNMEVLQK  FFELDWFTTK  MQDGLINPEAAAK  IPEFSWYSPIK  EEIKNNHVHDVK  YGIHKENDYFVYK  DEAIALFHLFYYAK  FNIPSYDTQSNVVPK  DIFIYHEGERFPYK  DYDIEMNMDNYTNKK  YDDNGIPLTLEDNWMK  RGEVYFYFYQQLLAR  SDCHGFVVPAPYEVYPK  MRDEAIALFHLFYYAK  IGKDYDIEMNMDNYTNK  TFVQFLQKDHFEAFGQK  IGKDYDIEMNMDNYTNKK  GTEGGFPFQLFVFVYPFDNK  ILSFFQDVSQLNTDDEYYK  NSNEFVIFKEDSVPMTEIMK  MLDEGKVPFDMSEEFCYMPK  KILSFFQDVSQLNTDDEYYK  LNHSPFNVNIEVDSNVASDAVVK  MLDEGKVPFDMSEEFCYMPK  AINFVGNYWQDNADLYGEEVTK  FTPFAQRPDYYNLHTEENYER  AINFVGNYWQDNADLYGEEVTKDYQR  DLAPFESFVLDNKPLGFPLDRPVVDALFK  GKDLAPFESFVLDNKPLGFPLDRPVVDALFK |
|  | P09179 | Sex-specific storage-protein 1 | 87890/80000 | 6.78/6.5 | 67.3% | 5231 | EGILTGK  TVDAVVR  LMSVNDK  DGTVISLK  DFDVFMR  DGTVISLKK  LMSVNDKR  DLGMSNTSK  QFMEMYK  YMNVDVVK  QNNMVVATK  AAKDPVLWK  FADVMIYR  EQFSFPGVK  SEDIENLAR  MKELCIMK  VIHLTNLMK  TSDMTFMAR  EPMVNLDMK  LPSGDEMPVR  VCNIFTVFK  FADVMIYRK  DPVFWMIMK  KSEDIENLAR  VIHLTNLMKK  TVDTSEMVMMK  MLSYGQYNMDK  IRLPSGDEMPVR  EQFSFPGVKVEK  QMMDDVEMMIR  LVLGGLEIVGDDAK  SCDKYMNVDVVK  KMLSYGQYNMDK  DDLTYLDSDMLVK  LDMFELDSFMYK  TGMLLPTLDMTMMK  VLYYAKDFDVFMR  MKQMMDDVEMMIR  RLDMFELDSFMYK  LLDHILQPTMFEDIK  RGEIMMYANQQLLAR  VMESEMMPSGDGQTMVK  LNHHPFQVSIDVMSDK  TGMLLPTLDMTMMKDR  SSMDMQGFIPEYLSTR  GETFVHTNELQMEEAVK  INGGMFVYAFTAACFHR  RVMESEMMPSGDGQTMVK  SSMDMQGFIPEYLSTRR  RLNHHPFQVSIDVMSDK  LLDHILQPTMFEDIKEIAK  MCDVKPMMWNEPLETGYWPK  LMLPLGTIGGLEMQMYVIVSPVR  LNHHPFQVSIDVMSDKTVDAVVR  GLYLPAPYEIYPYFFVDSHVISK  TVDTSEMVMMKDDLTYLDSDMLVK  WSSCISTMPLGYPFDRPIDMASFFTSNMK |
|  | P20613 | Sex-specific storage-protein 2 | 83698/80000 | 6.04/6.5 | 58.1% | 2619 | LTNGLGK  AVEEFLK  LYFDGVK  IDFHDPK  SYEVFAR  FLDTYEK  ITDVKVDK  DFETFYK  KAVEEFLK  TFVQFLQK  NVDAVFVEK  ENDYFVYK  DHFEAFGQK  NSNEFVIFK  IVEYIVEFK  VLGAAPMPFDK  TGYYPLMLTK  SKNVDAVFVEK  NLEFSVFYDK  EDSVPMTEIMK  QYLKPYTQDK  DPAFYQLYNR  RVLGAAPMPFDK  MFMNMEVLQK  FFELDWFTTK  IPEFSWYSPIK  MQHGLINPEAAAK  YGIHKENDYFVYK  DYDIEMNMDNYTNKK  YDDNGIPLTLEDNWMK  RGEVYFYFYQQLLAR  IGKDYDIEMNMDNYTNK  TFVQFLQKDHFEAFGQK  IGKDYDIEMNMDNYTNKK  GTEGGFPFQLFVFVYPFDNK  ILSFFQDVSQLNTDDEYYK  NSNEFVIFKEDSVPMTEIMK  KILSFFQDVSQLNTDDEYYK  LNHSPFNVNIEVDSNVASDAVVK  MLDEGKVPFDMSEEFCYMPK  AINFVGNYWQDNADLYGEEVTK  FTPFAQRPDYYNLHTEENYER  AINFVGNYWQDNADLYGEEVTKDYQR |
| **197** | Q1HPP4 | Arylphorin | 83569/80000 | 5.7/6.5 | 74.1% | 3186 | LTNGLGK  LTAGQNK  AVEEFLK  LYFDGVK  IDFHDPK  SYEVFAR  VPNMYFK  FLDTYEK  ITDVKVDK  DFETFYK  KAVEEFLK  TFVQFLQK  NVDAVFVEK  ENDYFVYK  DHFEAFGQK  NSNEFVIFK  IVEYIVEFK  VLGAAPMPFDK  TGYYPLMLTK  TKNVDAVFVEK  NLEFSVFYDK  DIFIYHEGER  EDSVPMTEIMK  QYLKPYTQDK  DPAFYQLYNR  RVLGAAPMPFDK  MFMNMEVLQK  FFELDWFTTK  MQDGLINPEAAAK  IPEFSWYSPIK  EEIKNNHVHDVK  YGIHKENDYFVYK  DEAIALFHLFYYAK  FNIPSYDTQSNVVPK  DIFIYHEGERFPYK  DYDIEMNMDNYTNKK  YDDNGIPLTLEDNWMK  RGEVYFYFYQQLLAR  SDCHGFVVPAPYEVYPK  MRDEAIALFHLFYYAK  IGKDYDIEMNMDNYTNK  TFVQFLQKDHFEAFGQK  IGKDYDIEMNMDNYTNKK  GTEGGFPFQLFVFVYPFDNK  ILSFFQDVSQLNTDDEYYK  NSNEFVIFKEDSVPMTEIMK  MLDEGKVPFDMSEEFCYMPK  KILSFFQDVSQLNTDDEYYK  LNHSPFNVNIEVDSNVASDAVVK  MLDEGKVPFDMSEEFCYMPK  AINFVGNYWQDNADLYGEEVTK  FTPFAQRPDYYNLHTEENYER  AINFVGNYWQDNADLYGEEVTKDYQR  DLAPFESFVLDNKPLGFPLDRPVVDALFK  GKDLAPFESFVLDNKPLGFPLDRPVVDALFK |
|  | P09179 | Sex-specific storage-protein 1 | 87890/80000 | 6.78/6.5 | 67.3% | 5231 | EGILTGK  TVDAVVR  LMSVNDK  DGTVISLK  DFDVFMR  DGTVISLKK  LMSVNDKR  DLGMSNTSK  QFMEMYK  YMNVDVVK  QNNMVVATK  AAKDPVLWK  FADVMIYR  EQFSFPGVK  SEDIENLAR  MKELCIMK  VIHLTNLMK  TSDMTFMAR  EPMVNLDMK  LPSGDEMPVR  VCNIFTVFK  FADVMIYRK  DPVFWMIMK  KSEDIENLAR  VIHLTNLMKK  TVDTSEMVMMK  MLSYGQYNMDK  IRLPSGDEMPVR  EQFSFPGVKVEK  QMMDDVEMMIR  LVLGGLEIVGDDAK  SCDKYMNVDVVK  KMLSYGQYNMDK  DDLTYLDSDMLVK  LDMFELDSFMYK  TGMLLPTLDMTMMK  VLYYAKDFDVFMR  MKQMMDDVEMMIR  RLDMFELDSFMYK  LLDHILQPTMFEDIK  RGEIMMYANQQLLAR  VMESEMMPSGDGQTMVK  LNHHPFQVSIDVMSDK  TGMLLPTLDMTMMKDR  SSMDMQGFIPEYLSTR  GETFVHTNELQMEEAVK  INGGMFVYAFTAACFHR  RVMESEMMPSGDGQTMVK  SSMDMQGFIPEYLSTRR  RLNHHPFQVSIDVMSDK  LLDHILQPTMFEDIKEIAK  MCDVKPMMWNEPLETGYWPK  LMLPLGTIGGLEMQMYVIVSPVR  LNHHPFQVSIDVMSDKTVDAVVR  GLYLPAPYEIYPYFFVDSHVISK  TVDTSEMVMMKDDLTYLDSDMLVK  WSSCISTMPLGYPFDRPIDMASFFTSNMK |
|  | P20613 | Sex-specific storage-protein 2 | 83698/80000 | 6.04/6.5 | 58.1% | 2619 | LTNGLGK  AVEEFLK  LYFDGVK  IDFHDPK  SYEVFAR  FLDTYEK  ITDVKVDK  DFETFYK  KAVEEFLK  TFVQFLQK  NVDAVFVEK  ENDYFVYK  DHFEAFGQK  NSNEFVIFK  IVEYIVEFK  VLGAAPMPFDK  TGYYPLMLTK  SKNVDAVFVEK  NLEFSVFYDK  EDSVPMTEIMK  QYLKPYTQDK  DPAFYQLYNR  RVLGAAPMPFDK  MFMNMEVLQK  FFELDWFTTK  IPEFSWYSPIK  MQHGLINPEAAAK  YGIHKENDYFVYK  DYDIEMNMDNYTNKK  YDDNGIPLTLEDNWMK  RGEVYFYFYQQLLAR  IGKDYDIEMNMDNYTNK  TFVQFLQKDHFEAFGQK  IGKDYDIEMNMDNYTNKK  GTEGGFPFQLFVFVYPFDNK  ILSFFQDVSQLNTDDEYYK  NSNEFVIFKEDSVPMTEIMK  KILSFFQDVSQLNTDDEYYK  LNHSPFNVNIEVDSNVASDAVVK  MLDEGKVPFDMSEEFCYMPK  AINFVGNYWQDNADLYGEEVTK  FTPFAQRPDYYNLHTEENYER  AINFVGNYWQDNADLYGEEVTKDYQR |
| **198** | H9JTA2 | Uncharacterized protein | 74049/73000 | 6.31/6.4 | 30.5% | 498 | FGSDAGR  SISLDGK  FAGSFLR  VSNAISNR  LIFPDIR  LMIELDK  VETYSDFR  LIDIFMDTK  EVTSVVPTGAR  KVETYSDFR  IFIAPTLDENK  IDISELEIWR  SVDSSVTVPFER  AIEDMAVILPNGR  MPIVIPSNYTASDAEPEQR  IFIAPTLDENKRPLNFDEQR |
|  | Q27451 | Phenoloxidase subunit 1 | 79305/73000 | 6.25/6.4 | 26% | 402 | FGSDAGR  FAGTTIR  VSNAIGNR  GMDFTPR  LDSTVASR  LDFPNIR  LMIELDK  LTPYGNDR  EVSSVVPSGAR  LIDIFMGMR  AAIEEGYFPK  SDVSELETWR  SIDSSVTIPYER  FLQAIENMSVMLPNGR  MPIVIPSNYTASDTEPEQR  IFIAPTVDESGKPFSFDEQR |
| **225** | Q27309 | Vitellogenin | 203725/40000 | 6.85/6.5 | 17.6% | 2198 | ELPVDLK  EQQQGLFR  TQEQQLIGR  YQPTPNIDK  VFEIEIDGGR  STNYGHCHHR  FANEQDPVEVTK  GLISALQLDTSAHR  TLAHLQEGPSSGSAFK  AETTSTVHVHPHLYGK  ELPKFANEQDPVEVTK  LENPQHGNFNEQLPDPR  VAYHFGVPVGAEWTGTAHK  YQPTPNIDKVFEIEIDGGR  FSSQSQSTGGQTYPSPWQVGK  VIHDSQNNYDREQQQGLFR  MRDMPIATMAPGALSFYQPLK  EGPIYKAETTSTVHVHPHLYGK  AEVYSHVHMELISVDQDSGAEWPR  KMETDVTGDCETLYTVSPVASEWR  IDFSYFDRTYTVDIASPSGEARMR |
| **226** | Q27309* | Vitellogenin (light chain) | 40203/40000 | 6.85/6.8 | 62.3% | 1779 | ELPVDLK  EQQQGLFR  TQEQQLIGR  YQPTPNIDK  VFEIEIDGGR  STNYGHCHHR  VIHDSQNNYDR  FANEQDPVEVTK  GLISALQLDTSAHR  TLAHLQEGPSSGSAFK  AETTSTVHVHPHLYGK  LENPQHGNFNEQLPDPR  VAYHFGVPVGAEWTGTAHK  IVSLDFPTSVPVPQENLIK  YQPTPNIDKVFEIEIDGGR  FSSQSQSTGGQTYPSPWQVGK  VIHDSQNNYDREQQQGLFR  METDVTGDCETLYTVSPVASEWR |
| **237** | H9JP12 | Sex-specific storage-protein 1 | 88007/80000 | 6.78/6.8 | 44.08% | 1728 | EGILTGK  TVDAVVR  LMSVNDK  DGTVISLK  DFDVFMR  DGTVISLKK  DLGMSNTSK  FADVMIYR  EQFSFPGVK  SEDIENLAR  MKELCIMK  VIHLTNLMK  TSDMTFMAR  EPMVNLDMK  LPSGDEMPVR  VCNIFTVFK  KSEDIENLAR  MLSYGQYNMDK  IRLPSGDEMPVR  EQFSFPGVKVEK  LVLGGLEIVGDDAK  LDMFELDSFMYK  RLDMFELDSFMYK  RGEIMMYANQQLLAR  LLDHILQPTMFEDIK  LNHHPFQVSIDVMSDK  SSMDMQGFIPEYLSTR  INGGMFVYAFTAACFHR  GETFVHTNELQMEEAVK  LLDHILQPTMFEDIKEIAK  GLYLPAPYEIYPYFFVDSHVISK |
| **239** | O97158 | Transferrin | 77156/80000 | 6.89/7 | 39.4% | 1032 | VLGLSDK  AIDYLNK  ALSTFFTK  YLCVDGSK  IPLTMLMK  AMSVFAFSR  VTLDCIPAR  TPNYAVAVVK  LCVTSNVALAK  TDEEPDAPFR  NNNVIFNNAAK  SIHDVISSCGIA  IHYVADNIPIK  DLPIDNLDQLK  ACQDMVDIPTK  GLATVENLDFEK  IPNQDFVVFQEYR  YVLHPVFHEVYGEK  SVQDNGSDLASVDDMR  DTLSNYPTCNIAMAPSR  KFFGLPVGTTPASPSNENPDEYR |
| **242** | Q27309** | Vitellogenin (heavy chain) | 161327/160000 | 6.85/6.8 | 26.8% | 2062 | VESGAEK  VSGSFDR  VFSVDSK  TSNNIIK  LVSAGINK  AIYDGQR  YGQSGEIR  AVLFSILR  VEISTYLR  LQFNIPFK  SYEYPLSR  VFAPYLEGK  YYEANMIK  NTAEPYPVR  IDFSYFDR  VQGMLFAGTK  SDMWMIFR  NDLSPNSETR  QIQSWIENK  FVVFTQDYR  DQQEIYISYK  GNWHELAILSR  DGVTQAGTLPAFK  GQNPEVSGIPSVK  NTIEASFSSAER  DSLVAISQDPATK  SAILSAAELQHPR  QIMTQFFNFAR  YTTELENHPLAK  KGQNPEVSGIPSVK  TYTVDIASPSGEAR  AQVYIQAIGNLGHR  ALATQQAYYPEYK  LIASMSTEQLSQTSR  YLVTNEEFGYQHSFK  ILQDIAQQLQNPNNMPK  DMPIATMAPGALSFYQPLK |
| **243** | Q27309** | Vitellogenin (heavy chain) | 161327/160000 | 6.85/7 | 24.1% | 1921 | VSGSFDR  TSNNIIK  LVSAGINK  AIYDGQR  YGQSGEIR  AVLFSILR  VEISTYLR  DSVFAPHPK  LQFNIPFK  SYEYPLSR  VFAPYLEGK  YYEANMIK  NTAEPYPVR  YYEANMIK  VQGMLFAGTK  IDFSYFDR  VQGMLFAGTK  SDMWMIFR  NDLSPNSETR  FVVFTQDYR  DQQEIYISYK  GNWHELAILSR  DGVTQAGTLPAFK  NTIEASFSSAER  GQNPEVSGIPSVK  DSLVAISQDPATK  SAILSAAELQHPR  KGQNPEVSGIPSVK  YKEPAVIHFQSK  TYTVDIASPSGEAR  ALATQQAYYPEYK  LIASMSTEQLSQTSR  YLVTNEEFGYQHSFK  ILQDIAQQLQNPNNMPK  DMPIATMAPGALSFYQPLK |
| **244** | Q27309** | Vitellogenin (heavy chain) | 161327/160000 | 6.85/7.1 | 27.1% | 1835 | VSGSFDR  TSNNIIK  LVSAGINK  AIYDGQR  YGQSGEIR  AVLFSILR  APFGMHFK  VEISTYLR  DSVFAPHPK  LQFNIPFK  SYEYPLSR  VFAPYLEGK  NTAEPYPVR  YYEANMIK  VQGMLFAGTK  IDFSYFDR  SDMWMIFR  QIQSWIENK  FVVFTQDYR  DQQEIYISYK  GNWHELAILSR  DGVTQAGTLPAFK  NTIEASFSSAER  GQNPEVSGIPSVK  DSLVAISQDPATK  SAILSAAELQHPR  YTTELENHPLAK  QIMTQFFNFAR  YKEPAVIHFQSK  TYTVDIASPSGEAR  AQVYIQAIGNLGHR  ALATQQAYYPEYK  LIASMSTEQLSQTSR  YLVTNEEFGYQHSFK  ILQDIAQQLQNPNNMPK  DMPIATMAPGALSFYQPLK |
| **245** | Q27309** | Vitellogenin (heavy chain) | 161327/160000 | 6.85/6.8 | 30.7% | 2323 | VSGSFDR  VFSVDSK  TSNNIIK  VPVTCQP  LVSAGINK  AIYDGQR  YGQSGEIR  AVLFSILR  VEISTYLR  LQFNIPFK  SYEYPLSR  VFAPYLEGK  YYEANMIK  NTAEPYPVR  VQGMLFAGTK  IDFSYFDR  SDMWMIFR  IQHVQVTCK  QIQSWIENK  FVVFTQDYR  DQQEIYISYK  GNWHELAILSR  DGVTQAGTLPAFK  NTIEASFSSAER  GQNPEVSGIPSVK  DSLVAISQDPATK  SAILSAAELQHPR  YTTELENHPLAK  QIMTQFFNFAR  KGQNPEVSGIPSVK  YKEPAVIHFQSK  TYTVDIASPSGEAR  AQVYIQAIGNLGHR  ALATQQAYYPEYK  LIASMSTEQLSQTSR  YLVTNEEFGYQHSFK  ILQDIAQQLQNPNNMPK  HDAFVLEEILPTLAADLK  LYEYLGYWYTEANPLK  FIDDSYDEDNDIGTFVISHIGSEDSLLPK |
| **247** | Q1HPS1 | ML-domain containing secreted protein | 17360/17000 | 6.28/7.2 | 27.9% | 324 | SVPELSK  AGTNYIYK  IHTEDDQK  NTDQHIVIK  FTPEKDVLDLK |
| **250** | Q1HQ02 | Ferritin | 26245/25000 | 6.75/7 | 49.8% | 843 | ALASLYLK  FITENNGK  LSDDSWEK  SLAGHTSDLK  ALASLYLKR  KLSDDSWEK  DNHVVANELK  MDFSSHTTLK  SLAGHTSDLKR  TLSLPHCSAYYGQFK  DLSLAVYLFDEYLQK  NSDLLHDAEITQYIEEEFVSQQADTIR |
| **259** | Q1HPN7 | Fructose-bisphosphate aldolase | 39971/40000 | 8.38/7.7 | 46.4% | 1376 | ATVTALLR  ALQASVLR  IAQAIVAPAK  TYTPNDVAR  YASICQSQR  VTEVVLAAVYK  YVAGSIPSLAASK  ADDGTPLVSLLEK  GILAADESTGTMGK  RPWVLTFSYGR  TENILAGQQELIK  LQDIGVENTEENR  TENILAGQQELIKR  NTPSYQAIQENANVLAR  IVPIVEPEVLPDGEHDLDR |
| **263** | H9ITY5 | Probable medium-chain specific acyl-CoA dehydrogenase. mitochondrial isoform X2 | 46461/45000 | 5.91/7.5 | 44.1% | 856 | ALQELAR  TFGVPIAR  IAMGAFDK  NTVMASVAK  GITFEDVR  NTVMASVAK  AFTGFIVER  EILTAATQTN  TGEYPWPVVK  ENVLIGEGAGFK  DEIIPVAGQYDK  TRPPVAAGATGLAQR  IYQIYEGTSQIQR  HQAVAFMLADMAIGVETAR  AATDAVQVFGGNGFNTEYPVEK  LIDEPLVAAYGVTEPGAGSDVAGIK |
| **266** | A7BEX9 | Imaginal disk growth factor | 48362/50000 | 7.64/7.2 | 52.8% | 1734 | FGTYAFR  EGFTALVR  LINPNQQK  VLCYYDSR  IVLSIATFGR  TFGTTPVDEK  STWGSLWHGIK  YNLLLESQQAR  GLCTGDKYPILR  LVSLNENLDIDR  TEGLLSYPEVCGK  ESEHREGFTALVR  EADYTAPIYTPQNR  NLGGVAIVDLSLDDFR  LVSLNENLDIDRTHDNYR  NPLQNADAAVTYWLTSGAPSQK  LPDDNGEGGIWVSYEDPDTAGQK  LDADSEIAGVPPIHTDGPGEAGPYVK  TFGTTPVDEKESEHREGFTALVR |
| **269** | Q68AP5 | Catalase | 57092/55000 | 8.11/8.2 | 67.5% | 2149 | SGAPVGIK  GIPDGYR  VFESIGK  TAIQTVGK  VAAGLAPYK  QVFDDAAK  DLYNSIAK  VFESIGKR  VAVSNYQR  LVLDRNPK  DSPGFITTK  FNPFDLTK  YNVGGDVDR  VAAGLAPYKK  DAAAFIQER  AIANIVDHLK  LFAYSDTHR  DPATDQLINYK  HMNGYGSHTFK  TLKDSPGFITTK  LVNSQGVGYWVK  IFSQVHPELGNK  DPATDQLINYKK  FSTVGGESGSADTVR  AGELASTDPDYSIR  DPTLFPSFIHTQK  IWPHAEYPLIPVGK  DPTLFPSFIHTQKR  GAGAFGYFEVTHDITK  LGANYLQIPVNCPYK  FSTVGGESGSADTVRDPR  NGPALLQDVNFLDEMSSFDRER  YNVGGDVDRYDSGQTEDNFSQATALYK  DGPQAIHNQDDCPNYFPNSFSGPQECPR |
| **270** | H9IYX7 | Bifunctional purine biosynthesis protein | 64577/60000 | 7.19/7.8 | 49.6% | 1977 | TGLLSLAK  ITAELFK  APEMLGGR  DLPSNAVR  GGNYCVLK  AALWWLR  HPSVLAMR  IALAHTNLR  DLIVATIALK  RHPSVLAMR  TIFGLTLEQK  EALSLPAAASFK  TIFGLTLEQKR  LSDSDQEDMKR  YTQSNSVCFAR  LSDSDQEDMKR  VALASDAFFPFR  TLHPAVHAGILAR  QYSPGQAQLTLR  DGQVIGIGAGQQSR  NAGLTVQDVSDITR  KQYSPGQAQLTLR  IDPTYEPSLMEQK  ELKEALSLPAAASFK  VTVVCDPADYDAVVK  EVSDGVIAPGYSPEALK  YGMNPHQKPAQVFTTR  VALASDAFFPFRDNIDR  SLSECGLQLIASGGTATALR  AFTHTSDYDLAISDYFR  AFTHTSDYDLAISDYFRK |
| **275** | H9IYX7 | Bifunctional purine biosynthesis protein | 64577/62000 | 7.19/7.5 | 49.2% | 1193 | TGLLSLAK  ITAELFK  APEMLGGR  DLPSNAVR  GGNYCVLK  AALWWLR  IALAHTNLR  DLIVATIALK  TIFGLTLEQK  EALSLPAAASFK  YTQSNSVCFAR  VALASDAFFPFR  TLHPAVHAGILAR  DGQVIGIGAGQQSR  NAGLTVQDVSDITR  IDPTYEPSLMEQK  ELKEALSLPAAASFK  VTVVCDPADYDAVVK  EVSDGVIAPGYSPEALK  YGMNPHQKPAQVFTTR  VALASDAFFPFRDNIDR  SLSECGLQLIASGGTATALR  AFTHTSDYDLAISDYFR  NAGLTVQDVSDITRAPEMLGGR  MSSFGDFVALSDPCDVSTATIISR |
| **276** | H9IZ23 | Pyruvate kinase | 68697/55000 | 9/7.2 | 27.8% | 941 | SAHLLSK  NVAVLEK  NGAALHEIR  QGSGFTNTVR  EAEAVIWHR  GDLGIEIPPEK  LTTSSDYQEK  MMETGMNVAR  NITNVVKPGNR  GNADTIYVDYK  LSGIICTIGPASR  LGSPFSLAIALDTK  GFVHPGDNAVVVTGWK  GVNLPGIPVDLPAVSEK  KGVNLPGIPVDLPAVSEK  GVLPIVYQEPTASDWLK |
|  | Q68AP5 | Catalase | 57092/55000 | 8.11/7.2 | 22% | 362 | GIPDGYR  VFESIGK  DLYNSIAK  VAVSNYQR  FNPFDLTK  LVNSQGVGYWVK  AGELASTDPDYSIR  IWPHAEYPLIPVGK  GAGAFGYFEVTHDITK  FSTVGGESGSADTVRDPR |
|  | H9J8X4 | Glucose-6-phosphate 1-dehydrogenase | 56942/55000 | 6.86/7.2 | 28% | 288 | VFTPILK  EPFGTEGR  DNLLPHNTK  IDYEFLNR  VQPGEALYLK  EMVQNLMTIR  GGYFDDFGIIR  IYPTIWYLYR  LILDVFTGTQMHFVR  LSNHLAGLFIEEQIYR  DLEDNHIKPVPYVYGSR |
| **279** | O97158 | Transferrin | 77156/80000 | 6.89/7.5 | 71.8% | 4089 | VLGLSDK  MVDIPTK  AIDYLNK  ALSTFFTK  SCHTGVNR  YLCVDGSK  IPLTMLMK  AMSVFAFSR  GHGAPELVVR  MNDHSISPK  VTLDCIPAR  TPNYAVAVVK  YEAVIVIHK  IIKPDQCIK  IPLTMLMKR  QCGSDSSPWK  LCVTSNVALAK  TDEEPDAPFR  KTPNYAVAVVK  NNNVIFNNAAK  SIHDVISSCGIA  SKVTLDCIPAR  DLPINNLDQLK  IHYVADNIPIK  GTSFNKMEDLR  LCSMCEHPER  GLATVENLDFEK  MECLNYVQQR  KGTSFNKMEDLR  CLAHNNGQVAFTK  TDLFNIYGEFLK  DRMECLNYVQQR  IPNQDFVVFQEYR  SVQDNGSDLASVDDMR  YVLHPVFHEVYGEK  CDYPDEFSGYVGALK  QLADAGSSNQPEWFTK  KYVLHPVFHEVYGEK  QADFVPVDPEDMYVAAK  DTLSNYPTCNIAMAPSR  DTLSNYPTCNIAMAPSR  TDEEPDAPFRYEAVIVIHK  DIRPILDCVQEASETDCLK  ACSWAARPWQGLIGHNDVLAK  FFGLPVGTTPASPSNENPDEYR  DFLSDVSIAHTPLSLAQLLDTR  SCHSSYGTFSGLDAPLYYLINK  KFFGLPVGTTPASPSNENPDEYR  SCHSSYGTFSGLDAPLYYLINKR  NFGDFFSGGSCLPGVDKPENNPSGDDVSSLKK |
| **283** | Q27309** | Vitellogenin (heavy chain) | 161327/160000 | 6.85/7.3 | 47.7% | 3470 | VSGSFDR  TSNNIIK  VPVTCQP  LVSAGINK  AIYDGQR  YGQSGEIR  AVLFSILR  APFGMHFK  VEISTYLR  SKVFSVDSK  DSVFAPHPK  LQFNIPFK  SYEYPLSR  VFAPYLEGK  YYEANMIK  NTAEPYPVR  IDFSYFDR  VQGMLFAGTK  FSFPSKDNK  IQHVQVTCK  SDMWMIFR  NDLSPNSETR  QIQSWIENK  FVVFTQDYR  QFKVPVTCQP  QIQSWIENKK  DQQEIYISYK  GNWHELAILSR  DGVTQAGTLPAFK  GQNPEVSGIPSVK  NTIEASFSSAER  DSLVAISQDPATK  SAILSAAELQHPR  YTTELENHPLAK  QIMTQFFNFAR  KGQNPEVSGIPSVK  YKEPAVIHFQSK  TYTVDIASPSGEAR  KDSLVAISQDPATK  AQVYIQAIGNLGHR  ALATQQAYYPEYK  SWHVVMQDESTQR  DGVLVINIEDDRIR  LIASMSTEQLSQTSR  YLVTNEEFGYQHSFK  ILQDIAQQLQNPNNMPK  HDAFVLEEILPTLAADLK  LYEYLGYWYTEANPLK  DMPIATMAPGALSFYQPLK  MRDMPIATMAPGALSFYQPLK  SESGQDLEIEIQPASGDSAYQVK  YGQSTHAVIYAQGYTYSSDWR  LTHKHDAFVLEEILPTLAADLK  VYNQDQVSIMFPVASGMPFIFK  AYELVANYFTGHQYQPYCSIDGTR  FRVEPLHPDQDQTLVHYSVWPYSASQK  FIDDSYDEDNDIGTFVISHIGSEDSLLPK |
| **284** | Q27309** | Vitellogenin (heavy chain) | 161327/160000 | 6.85/7.4 | 51.4% | 4638 | VSGSFDR  VFSVDSK  TSNNIIK  VPVTCQP  LVSAGINK  AIYDGQR  YGQSGEIR  AVLFSILR  APFGMHFK  VEISTYLR  SKVFSVDSK  DSVFAPHPK  LQFNIPFK  SYEYPLSR  VFAPYLEGK  NTAEPYPVR  YYEANMIK  VQGMLFAGTK  IDFSYFDR  FSFPSKDNK  SDMWMIFR  IQHVQVTCK  VPSCQSHCR  NDLSPNSETR  QIQSWIENK  FVVFTQDYR  GKFSFPSKDNK  DQQEIYISYK  GNWHELAILSR  DGVTQAGTLPAFK  GQNPEVSGIPSVK  NTIEASFSSAER  GQNPEVSGIPSVK  DSLVAISQDPATK  SAILSAAELQHPR  QIMTQFFNFAR  YTTELENHPLAK  EQPFLQYHTHK  KGQNPEVSGIPSVK  YKEPAVIHFQSK  TYTVDIASPSGEAR  KDSLVAISQDPATK  AQVYIQAIGNLGHR  KYTTELENHPLAK  ALATQQAYYPEYK  SWHVVMQDESTQR  DGVLVINIEDDRIR  LIASMSTEQLSQTSR  FLDYLRDSVFAPHPK  YLVTNEEFGYQHSFK  ILQDIAQQLQNPNNMPK  HDAFVLEEILPTLAADLK  LYEYLGYWYTEANPLK  DMPIATMAPGALSFYQPLK  MRDMPIATMAPGALSFYQPLK  SESGQDLEIEIQPASGDSAYQVK  YGQSTHAVIYAQGYTYSSDWR  LTHKHDAFVLEEILPTLAADLK  VYNQDQVSIMFPVASGMPFIFK  LFSFSESDLQQLSQYISEYMK  LFSFSESDLQQLSQYISEYMKK  AYELVANYFTGHQYQPYCSIDGTR  FRVEPLHPDQDQTLVHYSVWPYSASQK  FIDDSYDEDNDIGTFVISHIGSEDSLLPK  VAAIQSIFISHPTGEMMQAMAEMTHNDPSVEVR |
| **286** | Q27309** | Vitellogenin (heavy chain) | 161327/160000 | 6.85/7.5 | 40.6% | 3175 | VSGSFDR  VFSVDSK  TSNNIIK  VPVTCQP  LVSAGINK  AIYDGQR  YGQSGEIR  AVLFSILR  APFGMHFK  VEISTYLR  DSVFAPHPK  LQFNIPFK  SYEYPLSR  VFAPYLEGK  NTAEPYPVR  YYEANMIK  VQGMLFAGTK  IDFSYFDR  FSFPSKDNK  SDMWMIFR  QIQSWIENK  FVVFTQDYR  DQQEIYISYK  GNWHELAILSR  DGVTQAGTLPAFK  GQNPEVSGIPSVK  NTIEASFSSAER  DSLVAISQDPATK  SAILSAAELQHPR  QIMTQFFNFAR  YTTELENHPLAK  KGQNPEVSGIPSVK  YKEPAVIHFQSK  TYTVDIASPSGEAR  KDSLVAISQDPATK  AQVYIQAIGNLGHR  ALATQQAYYPEYK  SWHVVMQDESTQR  LIASMSTEQLSQTSR  YLVTNEEFGYQHSFK  ILQDIAQQLQNPNNMPK  LYEYLGYWYTEANPLK  DMPIATMAPGALSFYQPLK  MRDMPIATMAPGALSFYQPLK  SESGQDLEIEIQPASGDSAYQVK  YGQSTHAVIYAQGYTYSSDWR  VYNQDQVSIMFPVASGMPFIFK  AYELVANYFTGHQYQPYCSIDGTR  AIVMAHAPDHVEFSVSFQDMSPQYR  FRVEPLHPDQDQTLVHYSVWPYSASQK |
| **292** | Q69FX2 | Promoting protein | 17625/16000 | 8.37/8.8 | 68.8% | 863 | TGLFGLK  IDPCVNSR  LKTGLFGLK  LPTGNFEFK  KLPTGNFEFK  TQTLDFSLHIGK  EVDASACTVNEVR  VSFDFTPQFSTTK  LWNEDNESQMCCYR  NGAEIPFDALYNADACTLTSCPTEAGK |
| **295** | Q60GK5 | Glutathione S-transferase delta | 24269/23000 | 7.61/8.3 | 91.2% | 2143 | AVLLTAK  GLVNSMLK  GLVNSMLKK  ALNLNLNLK  AIITYLVNK  GSSLYPEDPK  VQEALQLLDK  STAPGYQEANEK  GSSLYPEDPKAR  LYFDIGTLYQR  AIITYLVNKYAK  TIDLYYVPGSAPCR  VQEALQLLDKFLEGQK  LVDLHHGEQLKPEYLK  FSDYFYPQVFAGAPADK  STAPGYQEANEKGLEAFK  FSDYFYPQVFAGAPADKAK  NEKVQEALQLLDKFLEGQK  LNPQHTVPTLVDDGLSIWESR  YVAGPNLTVADLSLIASVSSLEASDIDFKK |
| **309** | Q68AP5 | Catalase | 57092/55000 | 8.11/8 | 58.6% | 1430 | AGELASTDPDYSIR  AIANIVDHLK  DAAAFIQER  DLYNSIAK  DPTLFPSFIHTQK  DSPGFITTK  FNPFDLTK  FSTVGGESGSADTVRDPR  GAGAFGYFEVTHDITK  GIPDGYR  HMNGYGSHTFK  IWPHAEYPLIPVGK  LFAYSDTHR  LGANYLQIPVNCPYK  LVNSQGVGYWVK  NGPALLQDVNFLDEMSSFDR  NYFAEVEQIAFSPSNLVPGIEPSPDK  QVFDDAAK  SGAPVGIK  TAIQTVGK  TLKDSPGFITTK  VAAGLAPYK  VAVSNYQR  VFESIGK  YNVGGDVDRYDSGQTEDNFSQATALYK |
| **312** | G1UIS8 | Apolipophorin protein | 371420/73000 | 7.94/9 | 7.8% | 1511 | ISLDILK  FGYQDGK  VINGVPEK  QLDSEIR  LSISGPDGK  HVNNDALK  KISLDILK  VFLDALLR  ADGVVAISQK  VLEHFLGPK  HLENSLIEK  NDHACLGAVK  RVFLDALLR  TEEDTVVAVLK  AAAGLLDLPNLPK  SKTEEDTVVAVLK  TESPQAIYDSLVK  SDIIISNVNPNAGVK  DVYLGAGALAGAYCR  IQPQICAEEDDTR  DMQVLQSTLNVESK  AAVLEAFHADPCSPK  SVTNNVGANSASSFAQLVR  EQGKSDIIISNVNPNAGVK |
| **325** | C6L8Q2 | Putative acetyl transferase | 41580/40000 | 8.91/9.3 | 59.3% | 1082 | GIFIVGAK  TANDAGVFK  LGAQFGITR  TPFGTFGGVFR  ITAHLVHELR  AEIEPVSLTIK  RTPFGTFGGVFR  AGIPQEKPVLGINR  DEVDNYALQSQQK  NTSATELQTIATVGALK  ISVAGGVENMSQAPFAVR  LNVNGGATALGHPLGASGSR  LCGSGFQSIVNSAQDILTGAAK  LGAQFGITRDEVDNYALQSQQK  EGLVTAGTASGISDGAGALVLVSEEAAK  LTLNDVDLIEINEAFVAQTLSCAK |
|  | A0A0A0QY84 | Elongation factor 1-alpha | 50626/40000 | 9.24/9.3 | 41.7% | 1013 | GYVAGDSK  EAQEMGK  EVSSYIK  QLIVGVNK  TIEKFEK  RGYVAGDSK  LPLQDVYK  IGGIGTVPVGR  STTTGHLIYK  EHALLAFTLGVK  YYVTIIDAPGHR  MDSTEPPYSEPR  THINIVVIGHVDSGK  VETGVLKPGTIVVFAPANITTEVK  SVEMHHEALQEAVPGDNVGFNVK  IGYNPAAVAFVPISGWHGDNMLEPSTK  KIGYNPAAVAFVPISGWHGDNMLEPSTK |
| **328** | Q2F5T3 | ATP synthase subunit alpha | 59792/55000 | 9.21/9 | 51.2% | 1447 | APGIIPR  VGSAAQTK  ELIIGDR  STVAQIVK  ADLEETGR  LELAQYR  EPMQTGIK  VLSIGDGIAR  AVDSLVPIGR  GHLDKLDPSK  VVDALGNPIDGK  AAEISTILEER  HALIIYDDLSK  LYCIYVAIGQK  VSVREPMQTGIK  GIRPAINVGLSVSR  TALAIDTIINQQR  IVTDFLATFTQSQ  DGQITPESDASLKK  EAYPGDVFYLHSR  ITAFEKEFTQHIK  QVAGSMKLELAQYR  TGAIVDVPVGEQILGR  VVDALGNPIDGKGPIDTK  GMALNLEPDNVGVVVFGNDK  EVAAFAQFGSDLDAATQQLLNR |
| **332** | H9JP12 | Sex-specific storage-protein 1 | 88007/80000 | 6.78/9.3 | 20.02% | 679 | EGILTGK  TVDAVVR  LMSVNDK  DFDVFMR  DLGMSNTSK  QFMEMYK  FADVMIYR  EQFSFPGVK  SEDIENLAR  VIHLTNLMK  EPMVNLDMK  LPSGDEMPVR  VCNIFTVFK  MLSYGQYNMDK  IRLPSGDEMPVR  LVLGGLEIVGDDAK  GETFVHTNELQMEEAVK |
|  | Q1HPP4 | Arylphorin | 83569/80000 | 5.7/9.3 | 26.3% | 476 | AVEEFLK  SYEVFAR  FLDTYEK  DFETFYK  TFVQFLQK  NVDAVFVEK  ENDYFVYK  DHFEAFGQK  IVEYIVEFK  VLGAAPMPFDK  TGYYPLMLTK  NLEFSVFYDK  MFMNMEVLQK  FFELDWFTTK  MQDGLINPEAAAK  IPEFSWYSPIK  FNIPSYDTQSNVVPK  SDCHGFVVPAPYEVYPK |
